# Phylogeny and taxonomy of *Catenularia* and similar fungi with catenate conidia

**DOI:** 10.3897/mycokeys.81.67785

**Published:** 2021-06-11

**Authors:** Martina Réblová, Jana Nekvindová, Andrew N. Miller

**Affiliations:** 1 Czech Academy of Sciences, Institute of Botany, Průhonice 252 43, Czech Republic Czech Academy of Sciences, Institute of Botany Průhonice Czech Republic; 2 Department of Clinical Biochemistry and Diagnostics, University Hospital Hradec Králové, Hradec Králové 500 05, Czech Republic University Hospital Hradec Králové Hradec Králové Czech Republic; 3 Illinois Natural History Survey, University of Illinois Urbana-Champaign, Champaign, Illinois 61820, USA University of Illinois Urbana-Champaign Champaign United States of America

**Keywords:** angular conidia, basipetal chain, *
Chaetosphaeria
*, lignicolous, molecular systematics, phialidic conidiogenesis, 10 taxonomic novelties

## Abstract

The genus *Catenularia* (Chaetosphaeriaceae) was reviewed, and its relationships with morphologically similar fungi were evaluated using molecular and morphological data. Eleven species are accepted, four of which have been verified with molecular DNA data. The correct epithet ‘*cupulifera*’ is proposed for the type species *C. cupulifera***comb. nov.** Four other combinations are proposed, namely *C. catenulata***comb. nov.**, *C. elsikii***comb. nov.**, *C. minor***comb. nov.** and *C. novae-zelandiae***comb. nov.***Catenularia* is an uncommon fungus inhabiting mainly decaying bark, wood and bamboo culms of various hosts and shows a widespread geographical distribution. It is circumscribed for fungi with mononematous, macronematous, simple conidiophores with terminal monophialides, usually accompanied with capitate hyphae. The conidia are aseptate, brown, cuneiform to rounded-obconic with an angular outline, adhering in chains. The diagnostic values of taxonomic characteristics of capitate hyphae and conidia (i.e. colour, shape in transverse section, setulae and formation) at the generic level were evaluated. An account of morphology, taxonomy and phylogeny of species accepted in *Catenularia* is provided. Based on ribosomal DNA sequences, *Chalarodes obpyramidata***sp. nov.**, characterised by catenate, angular, hyaline conidia with apical setulae, is revealed as closely related to *Catenularia*. The new genus *Fuscocatenula***gen. nov.** is proposed for catenularia-like fungi having pigmented conidia with protracted maturation and round outline, with two species accepted, *F. submersa***comb. nov.** and *F. variegata***comb. nov.** A new species *Nawawia antennata***sp. nov.** is introduced and *Nawawia* is compared with morphologically similar taxa.

## Introduction

**Figure 1.**
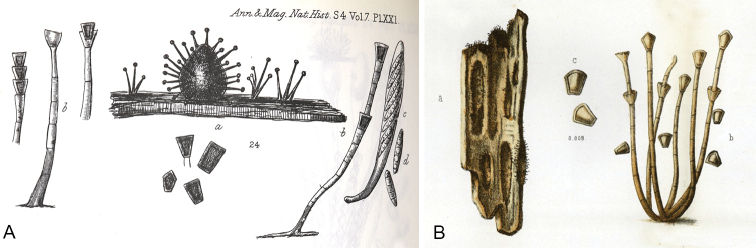
Illustrations of teleomorph and anamorph of *Catenularia cupulifera***A***Sphaeria cupulifera*: ascoma, ascus and ascospores with sporulating conidiophores (holotype, Berkeley and Broome 1871) **B***Psilonia cuneiformis*: conidiophores with conidia (holotype, Richon 1877).

*Catenularia* (Saccardo 1886) is one of the oldest genera classified in the Chaetosphaeriaceae. In April 1886, Saccardo introduced ‘*Catenularia* Grove in litt.’ with two species, ‘*C. simplex* Grove in litt.’ and *C. atra* (= *Spadicoides atra*, Hughes 1958), of which *C. simplex* is regarded as the type (Clements and Shear 1931). Grove (1886) intended the genus to be monotypic, and later that year published *Catenularia* again with *C. simplex* as the only species observed on wood in the United Kingdom. However, *C. simplex* has previously been described by Berkeley and Broome (1871) as the presumed but nameless conidial state of *Sphaeria cupulifera* on decaying elm roots also in the United Kingdom. The species was illustrated with pigmented conidiophores arising singly from ascomata and in tufts around them, with a funnel-shaped collarette and cuneiform, dark brown, aseptate conidia adhering in chains. The anamorph was named *Psilonia cuneiformis* by Richon (1877) based on a collection on wood in France and later transferred to the monotypic genus *Psiloniella* (Costantin 1888). Mason (1941) concluded that *P. cuneiformis* and *C. simplex* are conspecific and accepted *P. cuneiformis* in *Catenularia* with *C. simplex* listed as a synonym. De Seynes (1886) and Booth (1958) confirmed that *S. cupulifera* (= *Chaetosphaeria cupulifera*, Saccardo 1883) and *C. cuneiformis* belong to the life cycle of the same species (Fig. 1). Booth (1958) noted that the conidiophores develop from the modified outer cells of the ascomatal wall and arise from hyphae at the ascomatal bases.

Linder (1933) erected *Haplochalara* based on *H. angulospora* for fungi morphologically similar to *Catenularia* and compared it with *Chalaropsis* and *Thielaviopsis* based on the similar pigmented, phialidic conidia in chains. Both latter genera are currently accepted in the Ceratocystidaceae (De Beer et al. 2014). Mason (1941) was the first to suggest the remarkable similarity of *H. angulospora* to *Catenularia* and transferred it to the latter genus.

Hughes (1965) presented the first comprehensive treatise of *Catenularia* and accepted four species. The genus was circumscribed for lignicolous hyphomycetes with simple, pigmented conidiophores arising solitary or in tufts, with dark stromatic cells around their bases, accompanied by capitate hyphae and with monophialidic conidiogenous cells extending percurrently. The conidia adhere in chains; they are aseptate, brown, cuneiform to rounded-obconic in side view, polygonal in transverse section with a small, circular, thin-walled, pale area at each corner. Capitate hyphae, a term coined by Hughes (1949), were originally proposed for sterile hyphae scattered among conidiophores of *Sporoschisma*. These are erect, brown, septate hyphae that extend percurrently and terminate into a paler, swollen apex. The apical cell bears a mucilaginous hyaline cap or pale coloured droplets that may disappear with age. Capitate hyphae also occur on the ascomatal wall of the teleomorphs.

Hughes (1965) did not accept the synonymy of *Catenularia* and *Haplochalara*. He considered capitate hyphae to be one of the main diagnostic features at the generic level, but which were missing in *H. angulospora*. Hughes (1965) excluded from *Catenularia* another nine species with ellipsoidal or globose, hyaline or slightly pigmented conidia, different conidiogenous cell morphology and modes of conidiogenesis. Some of these species have been reclassified and are currently attributed to genera such as *Chloridium*, *Exochalara*, *Gliomastix*, *Monilochaetes*, *Periconia*, *Spadicoides*, *Sporoschismopsis* and *Thielaviopsis* (Mangenot 1952; Booth 1957; Hughes 1958, 1965; Von Arx 1970; Holubová-Jechová and Hennebert 1972; Gams and Holubová-Jechová 1976; Schoknecht and Crane 1977; Rong and Gams 2000; Mbenoun et al. 2014; De Beer et al. 2014). Other authors did not follow such a narrow generic circumscription and several species without capitate hyphae were introduced in *Catenularia*, namely *C. catenulata* (Luo et al. 2019), *C. hughesii* (Sharma 1980), *C. kalakadensis* and *C. malabrica* (Subramanian and Bhat 1989), and *C. variegata* (Li et al. 2017). Admission of *C. variegata* in *Catenularia* introduced further heterogeneity into the genus. This species has a protracted maturation of conidia that are cuneiform or obovoid in the side view, but have round outline and lack typical corners with pore-like areas at the apex.

Species with the *Catenularia* morphotype have been named inconsistently as *Catenularia* or *Chaetosphaeria*. To date, 24 species and varieties have been referred to as *Catenularia* and six as their *Chaetosphaeria* counterparts (Berkeley and Broome 1871; Saccardo 1886; Linder 1933; Booth 1958; Hughes 1965; Sharma 1980; Holubová-Jechová 1982, 1983; Subramanian and Bhat 1989; Réblová and Seifert 2003; Li et al. 2017; Luo et al. 2019). They have a saprobic lifestyle and occur on decaying bark, wood or bamboo culms in terrestrial, less often freshwater habitats worldwide. Pound et al. (2019) published *Ch. elsikii*, a fossil species similar to the *Catenularia* anamorph of *Ch. novae-zelandiae*. After the abolishment of dual nomenclature and subsequent changes to the International Code of Nomenclature for algae, fungi, and plants (ICN; McNeill et al. 2012), *Catenularia* has never been formally accepted as a holomorphic genus, along with the correct taxonomic treatment of its type species.

The characteristics of conidia, conidiogenous cells, conidiophores and the mode of conidiogenesis are the main diagnostic traits that distinguish genera of the Chaetosphaeriaceae, while their teleomorphs are usually morphologically uniform. Among members of the family, *Catenularia*, *Nawawia* (Marvanová 1980) and *Phialosporostilbe* (Mercado Sierra and Mena Portales 1985) share a basic pattern of turbinate to obpyramidal, angular and aseptate conidia. The conidia of *Catenularia* are brown and without setulae, conidia of the latter genera are hyaline with several setulae at the apex, occasionally also at the base. *Nawawia* contains species with mononematous conidiophores, terminal monophialides elongating percurrently, and conidia aggregated in heads. In contrast, *Phialosporostilbe* has synnematous conidiophores associated with setae, terminal monophialides and conidia aggregated in heads, rarely in chains (Mercado Sierra and Mena Portales 1985; Sureshkumar et al. 2005). *Nawawia* and *Phialosporostilbe* are saprobes on decaying plant material, often submerged in freshwater, occasionally isolated from soil (e.g. Marvanová 1980; Mercado Sierra and Mena Portales 1985; Bhat and Kendrick 1993; Mel’nik and Hyde 2006; Wu and Zhang 2009; Goh et al. 2014). In characters of conidia, they closely resemble *Chalarodes* (McKenzie 1991) and *Obeliospora* (Nawawi and Kuthubutheen 1990), whose systematic placement remains unexplored. The genus *Chalarodes* includes fungi inhabiting decaying palm leaves, and is widespread in Australasia (McKenzie 1991). The conidia adhere in basipetal chains and are borne on terminal monophialides on mononematous conidiophores. The colonies of *Obeliospora* are composed of dark, acute setae accompanied by short, monilioid conidiophores with doliiform conidiogenous cells and conspicuous cup-shaped collarettes. The genus accommodates species that thrive on submerged wood or plant litter in freshwater biotopes, occasionally they occur in terrestrial habitats, in South America and Southeast Asia (Nawawi and Kuthubutheen 1990; Kuthubutheen and Nawawi 1994; Wu and Mckenzie 2003; Cantillo-Pérez et al. 2018).

This study is based on nuc rDNA sequences combined with a comparative analysis of phenotypic data. It aims to evaluate the generic concept of *Catenularia* and its relationships with morphologically similar taxa. Another aim is to assess whether phenotypic characteristics such as the presence or absence of capitate hyphae and selected conidial features (i.e. colour, shape in transverse section, setulae and formation at the tip of the conidiogenous cell) are congruent with phylogenetic relationships.

## Materials and methods

### Fungal strains, morphology and DNA extraction and PCR amplification

Specimens of *Catenularia*, *Chalarodes*, *Nawawia* and *Sporoschisma* were collected in various localities in temperate and tropical geographical areas in Cuba, Czech Republic, France, Belgium, Martinique, New Zealand, Slovak Republic and Thailand. Other specimens were obtained from the Canadian National Mycological Herbarium (DAOM, Ottawa, Canada), Farlow herbarium (FH, Harvard University, Cambridge, Massachusetts, USA), New Zealand Fungarium (PDD, Auckland, New Zealand), Herbarium of the National Museum (PRM, Prague, Czech Republic), and Herbarium of the Naturhistorisches Museum Wien (W, Vienna, Austria). Holotypes and specimens (as dried voucher specimens) were deposited at PDD and Herbarium of the Institute of Botany (PRA, Průhonice, Czech Republic). Fungal novelties were registered in MycoBank.

For morphological study, isolation and cultivation we follow Réblová et al. (2021a) and references cited therein. Axenic cultures were derived from freshly collected material. Strains were inoculated on potato-carrot agar (PCA) (Crous et al. 2019).

Protocols for the DNA extraction and PCR amplification followed Huhndorf et al. (2004), Hustad and Miller (2015) and Réblová et al. (2020). Automated sequencing was carried out by Eurofins GATC Biotech Sequencing Service (Cologne, Germany), Ottawa Research and Development Centre, Biodiversity (Mycology and Microbiology), Agriculture and Agri-Food Canada (Ottawa, Ontario, Canada) and the Roy J. Carver Biotechnology Center at the University of Illinois Urbana-Champaign (Champaign, Illinois, USA). Raw sequence data were analysed using Sequencher v.5.4.6 (Gene Codes Corp., USA, Michigan, Ann Arbor).

### Alignments and phylogenetic analyses

In order to assess relationships of *Catenularia* with similar fungi, sequences of the internal transcribed spacer region (ITS1-5.8S-ITS2) (ITS) of the nuclear rRNA cistron and the large subunit 28S rDNA gene (28S) (ca. 1800 base pairs at the 5′-end) were analysed. Isolates, their sources and GenBank accession numbers of sequences generated in this study and those retrieved from GenBank and published in other studies (Réblová and Winka 2000, 2001; Fernández et al. 2006; Somrithipol et al. 2008; Shenoy et al. 2010; Magyar et al. 2011; Crous et al. 2012; Hashimoto et al. 2015; Hernández-Restrepo et al. 2016, 2017; Liu et al. 2016; Lu et al. 2016; Ma et al. 2016; Yang et al. 2018; Lin et al. 2019; Luo et al. 2019; Vu et al. 2019; Réblová et al. 2020, 2021a, b) are listed in the Suppl. material 1: Table S1.

Consensus secondary structure (2D) models for the ITS1 and ITS2 for members of the Chaetosphaeriaceae were built using the Ppfold program v.3.0 (Sukosd et al. 2012). The obtained 2D consensus models were further improved using the program Mfold (Zuker 2003) and RNAfold web server through the ViennaRNA Web Services (Gruber et al. 2015) and adjusted manually if necessary. The predicted 2D RNA structures were obtained in a dot bracket notation and were visualised and drawn using the program VARNA: Visualisation Applet for RNA (Darty et al. 2009).

Sequences were aligned manually in Bioedit v.7.1.8 (Hall et al. 1999). Consensus 2D structure models for the ITS1 and ITS2 were used to compare nucleotides at homologous positions (in helices and loops) and construct a reliable multiple sequence alignment. A predicted 2D model of the 28S of *Saccharomyces cerevisiae* (Gutell et al. 1993) was used to improve the alignment of this gene. The models were highly consistent in all species.

The ITS and 28S datasets, for which we assumed rate heterogeneity, were evaluated using PartitionFinder2 (Lanfear et al. 2017), implemented in the CIPRES Science Gateway v.3.3 (Miller et al. 2010), to find the best partitioning scheme for our datasets and to select best-fit models under corrected Akaike information criteria. Phylogenetic reconstructions were performed using Bayesian Inference (BI) and Maximum Likelihood (ML) analyses through the CIPRES Science Gateway v.3.3. ML analysis was conducted with RAxML-HPC v.8.2.12 (Stamatakis 2014) with a GTRCAT approximation. BI analysis was executed in a likelihood framework as implemented in MrBayes v.3.2.6 (Huelsenbeck and Ronquist 2001). The phylogenetic analyses were performed as described in Réblová et al. (2021a).

The conflict-free single locus data sets were concatenated and the ITS-28S alignment (deposited in TreeBASE) was subjected to the phylogenetic analysis. Ninety nucleotides (nt) at the 5′-end of 28S were excluded from the alignment because of the incompleteness in the majority of sequences. The full dataset consisted of 2386 characters including gaps (ITS = 612 characters; 28S = 1774) and 1038 unique character sites (RAxML). For the BI analysis, GTR+I+G model was selected for both partitions. *Tracylla aristata* and *T. eucalypti* (Tracyllales) were selected as outgroup taxa.

## Results

### Phylogenetic analyses

In the phylogenetic analysis of the combined ITS-28S sequences, we evaluated systematic placement of *Catenularia* in the Chaetosphaeriaceae and its relationships with morphologically similar taxa. The ML and BI trees were largely congruent; the ML tree is shown in Fig. 2. The Chaetosphaeriaceae included 49 well supported clades that correspond to individual genera or natural groups of species. The genus *Catenularia* was resolved as a monophyletic, strongly supported clade (95% ML, BS 1.0 PP) with four species, *C. angulospora*, *C. cubensis*, *C. minor* and *C. catenulata*. *Catenularia* resided in a statistically well supported clade at the base of the tree. This clade contained six other genera and natural groups of species, including *Exserticlava vasiformis* and *Stanjehughesia hormiscioides*, known to form capitate hyphae on ascomata of their teleomorphs. *Catenularia* was shown as a sister (95/1.0) to an unknown species of *Chalarodes*, described as *Cha. obpyramidata* below. Morphologically similar genera *Nawawia* and *Phialosporostilbe* were resolved as separate lineages. *Chaetosphaeria submersa*, superficially resembling *Catenularia*, was clustered in a distantly related clade containing *Phaeostalagmus*, and also *Ch. innumera* and another two *Chaetosphaeria* species with anamorphs with catenate conidia, i.e. *Chloridium clavaeforme* and *Ch. phaeophorum*.

**Figure 2.**
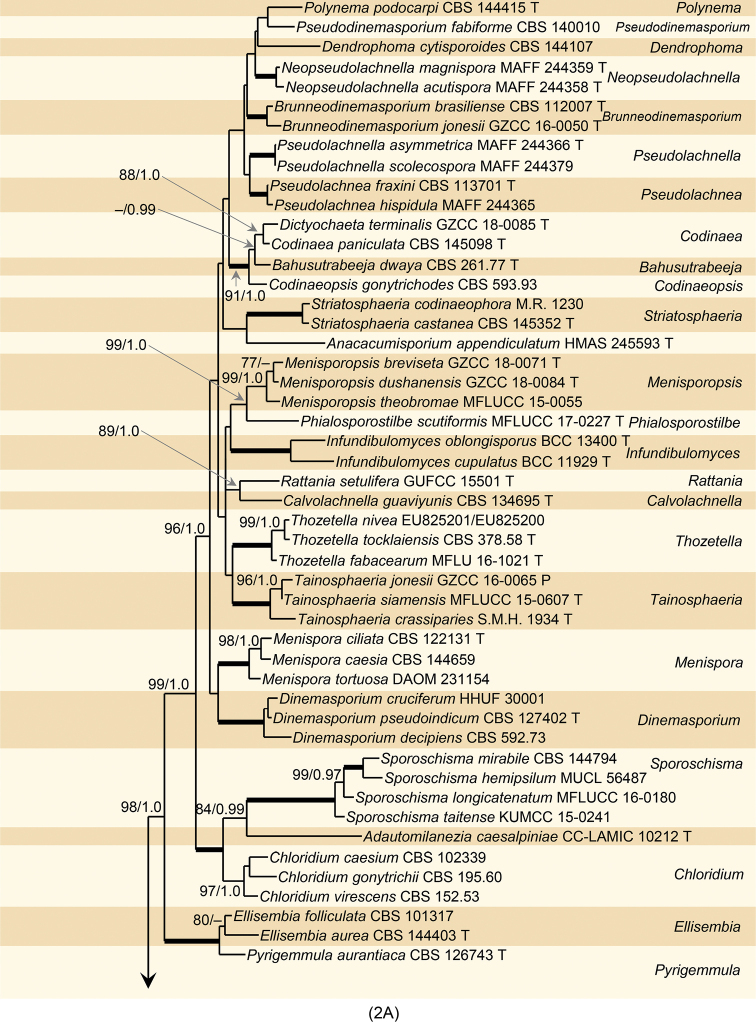
**A** Phylogenetic analysis of the combined ITS and 28S sequences of members of the Chaetosphaeriaceae. Species names given in bold are taxonomic novelties; T, E, I, N and P indicate ex-type, ex-epitype, ex-isotype, ex-neotype and ex-paratype strains; * holotype of *Chaetosphaeria trianguloconidia*; # *Catenularia cubensis fide*Luo et al. (2019). Thickened branches indicate branch support with ML BS = 100%, PP values = 1.0. Branch support of nodes ≥ 75% ML BS and ≥ 0.95 PP is indicated above and below branches **B** phylogenetic analysis of ITS and 28S of the Chaetosphaeriaceae (continued). For legend refer to (**A**). Abbreviation: p.p. after a genus name (*pro parte*).

**Figure 2.**
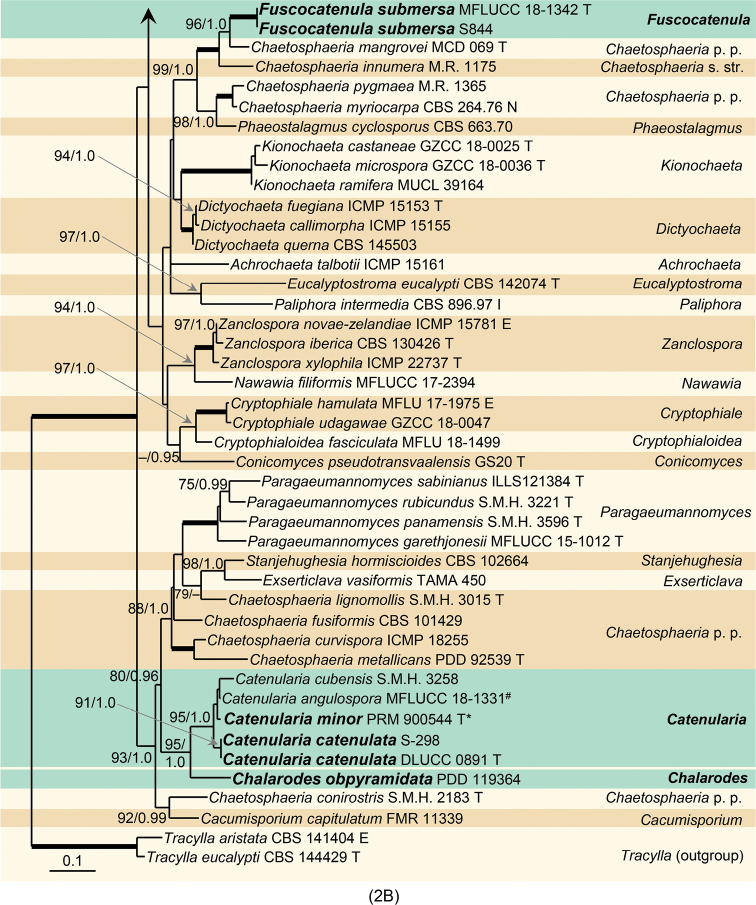
Continued.

### Taxonomy

#### Catenularia

Taxon classificationFungiChaetosphaerialesChaetosphaeriaceae

Grove, Syll. fung. 4: 303. 1886.

89E4CCBB-F476-5098-A900-F8C5DF46F1CD

Synonyms. Psiloniella Costantin, Mucéd. Simpl.: 25, 190. 1888.
Haplochalara Linder, Mycologia 25: 347. 1933.

##### Type species.

*Catenularia cupulifera* (Berk. & Broome) Réblová & A.N. Mill.

##### Emended description.

Colonies effuse, hairy to velutinous, brown, dark brown to black, mycelium partly immersed, partly superficial; composed of conidiophores, capitate hyphae and sometimes ascomata. ***Anamorph*.** Conidiophores macronematous, mononematous, solitary or in tufts, with dark stromatic hyphal cells around the bases, erect, straight or flexuous, unbranched, brown to dark brown, thick-walled, paler and thinner-walled towards the apex. Capitate hyphae scattered among the conidiophores, occasionally absent, erect, brown, extending percurrently, paler towards the apex, apical cell sterile, thin-walled, subhyaline to hyaline, slightly swollen, broadly rounded with a hyaline mucilaginous cap that may disappear with age. Conidiogenous cells integrated, terminal, monophialidic, extending percurrently, cylindrical, subcylindrical or somewhat lageniform, brown, conidia produced successively; collarettes cup- or funnel-shaped, brown, smooth or slightly roughened, margin entire or frayed. Conidia cuneiform, obclavate, rounded-obconic to broadly obovoid in side view, with an angular outline when viewed from above with 3–6 blunt corners, broadly rounded to flattened at the apex, truncate at the distinctive, hyaline basal hilum, with a small, circular, thin-walled, pore-like area visible in the cell wall at each corner, sometimes with a visible central pore at the base, aseptate, hyaline when young, fuscous, fulvous, brown to dark brown at maturity, thick-walled, smooth; formed singly, adhered in basipetal chains, occasionally in clusters. ***Teleomorph*.** Ascomata perithecial, non-stromatic, superficial, globose, subglobose to conical, papillate, glabrous occasionally with a powdery layer that disappears with age, sometimes covered with conidiophores and capitate hyphae. Ostiolar canal periphysate. Ascomatal wall carbonaceous, two-layered. Paraphyses persistent, branching, anastomosing, hyaline, longer than the asci. Asci unitunicate, short-stipitate, apical annulus non-amyloid, with eight ascospores. Ascospores fusiform, transversely septate, hyaline, smooth, without mucilaginous sheath or appendages.

##### Habitat and geographical distribution.

Saprobe on decaying bark, wood and bamboo culms of various hosts. Members of *Catenularia* have a worldwide distribution in temperate, subtropical and tropical geographic areas.

##### Notes.

Hughes (1965) considered capitate hyphae to be an important diagnostic characteristic of *Catenularia*. These structures have long escaped attention, and mycologists began to notice them only after they were described by Hughes (1949). We studied holotype material of several species and original descriptions and illustrations to examine and trace this character in *Catenularia*. Capitate hyphae have not been mentioned in the original descriptions of *C. cupulifera* (Berkeley and Broome 1871; Richon 1877; Grove 1886). In studying collections of this species, we observed a variation in the presence of capitate hyphae. In some specimens, capitate hyphae are abundantly present, but may be scarce and difficult to find in others. Revision of the holotypes of C. cuneiformis var. minor (Holubová-Jechová 1983) and *Ch. trianguloconidia* (Réblová and Seifert 2003) not only revealed that both fungi are conspecific, but also led to the discovery of capitate hyphae, although they were not mentioned in the protologues of either species. They are scattered among conidiophores and easy to overlook. Phylogenetic analysis of several *Catenularia* representatives with capitate hyphae (*C. cubensis* and *C. minor*) and those without them (*C. angulospora*, *C. catenulata*) provided compelling evidence to consider these species congeneric.

In this study, we present a taxonomic circumscription of *Catenularia* using molecular and phenotypic data. The generic concept has been emended and species with and without capitate hyphae are accepted in *Catenularia*. We were unsuccessful in obtaining *C. cupulifera* into axenic culture from fresh material. The available non-type strain CBS 419.80 of this species is a contaminant (In the Blast search, ITS and 28S sequences derived from this strain showed 100% identity with sequences of various strains of *Calycina citrina*.). Eleven species are accepted in *Catenularia* and listed below, four of which have been verified with molecular DNA data. Other species are accepted based on morphological similarity, but have to be confirmed as members of *Catenularia* by molecular data. So far, the teleomorph has been observed in *C. cubensis*, *C. cupulifera*, *C. minor* and *C. novae-zelandiae*. *Catenularia variegata* (Li et al. 2017) is excluded from *Catenularia* and transferred to a new segregate genus *Fuscocatenula* in this study. Disposition of *Catenularia* and morphologically similar taxa previously attributed to the genus is presented in Table 1.

**Table 1.** Species of *Catenularia*: accepted species (in bold) and disposition of morphologically similar taxa previously treated in *Catenularia* and *Chaetosphaeria*.

Name in *Catenularia* and *Chaetosphaeria*	Current name	Current classification	Reference
*Catenularia angulospora* (Linder) E.W. Mason*	***Catenularia angulospora*** (Linder) E.W. Mason	Chaetosphaeriales	Mason (1941)
*C. antarctica* Henn.*	*Periconia antarctica* (Henn.) S. Hughes	Pleosporales	Hughes (1965)
*C. atra* (Corda) Sacc.*	*Spadicoides atra* (Corda) S. Hughes	Xenospadicoidales	Hughes (1958)
*C. cubensis* Hol.-Jech.	***Catenularia cubensis*** Hol.-Jech.	Chaetosphaeriales	Holubová-Jechová (1982)
C. cuneiformis var. cuneiformis (Richon) E.W. Mason	***Catenularia cupulifera*** (Berk. & Broome) Réblová & A.N. Mill.	Chaetosphaeriales	Mason (1941), this study
C. cuneiformis var. minor Hol.-Jech.	***Catenularia minor*** (Hol.-Jech.) Réblová & A.N. Mill.	Chaetosphaeriales	Holubová-Jechová (1983), this study
*C. echinata* Wakker*	*Thielaviopsis ethacetica* Went	Microascales	De Beer et al. (2014)
*C. elasticae* Koord.*	*Gliomastix elasticae* (Koord.) Crane & Schoknecht	Hypocreales	Schoknecht and Crane (1977)
*C. fuliginea* Saito*	*Wallemia sebi* (Fr.) Arx	Wallemiales	Von Arx (1970)
C. fuliginea var. lunzinensis Szilv.*	Catenularia fuliginea var. lunzinensis Szilv.	unknown	Von Szilvinyi (1941)
*C. guadalcanalensis* Matsush.	*Monilochaetes guadalcanalensis* (Matsush.) I.H. Rong & W. Gams	Glomerellales	Rong and Gams (2000)
*C. heimii* F. Mangenot*	*Chloridium clavaeforme* (Preuss) W. Gams & Hol.-Jech.	Chaetosphaeriales	Gams and Holubová-Jechová (1976)
*C. hughesii* N.D. Sharma	***Catenularia angulospora*** (Linder) E.W. Mason	Chaetosphaeriales	Sharma (1980)
*C. kalakadensis* Subram. & Bhat	***Catenularia kalakadensis*** Subram. & Bhat	Chaetosphaeriales	Subramanian and Bhat (1989)
*C. longispora* S. Hughes	***Catenularia longispora*** S. Hughes	Chaetosphaeriales	Hughes (1965)
*C. macrospora* S. Hughes	***Catenularia macrospora*** S. Hughes	Chaetosphaeriales	Hughes (1965)
*C. malabarica* Subram. & Bhat	***Catenularia malabarica*** Subram. & Bhat	Chaetosphaeriales	Subramanian and Bhat (1989)
*C. megalospora* Speg.*	*Catenularia megalospora* Speg.	unknown	Spegazzini (1898)
*C. piceae* M.B. Ellis	*Exochalara longissima* (Grove) W. Gams & Hol.-Jech.	Helotiales	Gams and Holubová-Jechová (1976)
*C. pidopliczkoi* (Zhdanova) M.A. Litv.	*Haplochalara pidoplitschkoi* Zhdanova	unknown	Litvinov (1967)
*C. simmonsii* Morgan-Jones	*Sporoschismopsis simmonsii* (Morgan-Jones) Hol.-Jech. & Hennebert	Glomerellales	Holubová-Jechová and Hennebert (1972)
*C. simplex* Grove	***Catenularia cupulifera*** (Berk. & Broome) Réblová & A.N. Mill.	Chaetosphaeriales	Saccardo (1886), this study
*C. variegata* H.H. Li & X.G. Zhang	*Fuscocatenula variegata* (H.H. Li & X.G. Zhang) Réblová & A.N. Mill.	Chaetosphaeriales	Li et al. (2017), this study
*C. velutina* Syd. & P. Syd.*	*Catenularia velutina* Syd. & P. Syd.	unknown	Sydow and Sydow (1914)
*Chaetosphaeria catenulata* Z.L. Luo, K.D. Hyde & H.Y. Su	***Catenularia catenulata*** (Z.L. Luo, K.D. Hyde & H.Y. Su) Réblová & A.N. Mill.	Chaetosphaeriales	Luo et al. (2019), this study
*Ch. cubensis* Hol.-Jech.	***Catenularia cubensis*** Hol.-Jech.	Chaetosphaeriales	Holubová-Jechová (1983), this study
*Ch. cupulifera* (Berk. & Broome) Sacc.	***Catenularia cupulifera*** (Berk. & Broome) Réblová & A.N. Mill.	Chaetosphaeriales	Berkeley and Broome (1871), this study
*Ch. elsikii* M.J. Pound et al.	***Catenularia elsikii*** (M.J. Pound et al.) Réblová & A.N. Mill.	Chaetosphaeriales	Pound et al. (2019), this study
*Ch. novae-zelandiae* S. Hughes & Shoemaker	***Catenularia novae-zelandiae*** (S. Hughes & Shoemaker) Réblová & A.N. Mill.	Chaetosphaeriales	Hughes (1965), this study
*Ch. trianguloconidia* Réblová & Seifert	***Catenularia minor*** (Hol.-Jech.) Réblová & A.N. Mill.	Chaetosphaeriales	Réblová and Seifert (2003), this study

Notes: Species marked with an asterisk (*) were excluded from the genus by Hughes (1965). Note that some species listed among currently accepted names are included more than once due to the revealed synonymy.

*Haplochalara* (Linder 1933) and *Psiloniella* (Costantin 1888) are accepted as generic synonyms of *Catenularia*. The systematic placement of *H. pidoplitschkoi* (Litvinov 1967) is unknown. The species was characterised by dematiaceous, erect, simple conidiophores producing ellipsoidal, hyaline conidia that accumulate in slimy droplets and formation of dark chlamydospores in culture. Based on these characteristics, the species shows affinity to *Chloridium* (Gams and Holubová-Jechová 1976) and would be better placed in this genus.

### Key to *Catenularia* species

**Table:** 

1	Capitate hyphae present	**2**
–	Capitate hyphae absent or this character is unknown	**7**
2	Conidia 5.5–8.5 μm long, 3.5–5.5 μm wide at the apical end, 1.5–2 μm wide at base, with three bluntly rounded corners	***C. cubensis***
–	Conidia 9 μm and longer	**3**
3	Conidia up to 13.5 μm long and up to 11.5 μm wide	**4**
–	Conidia 13.5 μm and longer, wider than 11.5 μm	**5**
4	Conidia (9–)10.5–13.5 μm long, 7–9.5 μm wide at the apical end, 3.5–4.5 μm wide at the basal hilum, with (3–)4(–5) blunt corners	***C. cupulifera***
–	Conidia (6.5–)7.5–10.5(–13) μm long, 6.5–11.5 μ wide at the apical end, 1.5–2.5 μm wide at the base, with 3–5 blunt corners	***C. minor***
5	Conidia 11.5–17.5 μm long, 14.5–18.5 μm wide at the apical end, 4–5.5 μm wide at the base, with 4–5 blunt corners	***C. novae-zelandiae***
–	Conidia longer than 17.5 μm	**6**
6	Conidia 21–28 μm long, 19–28 μm wide at the apical end, 4–7 μm wide at the base, with (3–)4(–5) blunt corners	***C. macrospora***
–	Conidia 27–45 μm long, 16.8–24 μm wide at the apical end, 7–10 μm wide at the base, with three blunt corners	***C. longispora***
7	Conidia up to 9 μm long	**8**
–	Conidia longer than 9 μm	**9**
8	Conidia 6–8(–9) μm long, 4.5–6(–7) μm wide at the apical end, ca. 2 μm wide at the base, with three blunt corners	***C. angulospora***
–	Conidia up to 8 μm long, 6–7 μm wide at the apical end, 1.5–3.5 μm side at the base, with six corners	***C. kalakadensis***
9	Conidia 13–15 μm long, 12–14 μm wide at the apical end, with 3–4 corners	***C. catenulata***
–	Conidia wider than 15 μm	**10**
10	Conidia 12–18 μm long, 18–21 μm wide, 3–4 μm wide at the base, with 4–5 corners	***C. malabrica***
–	Conidia 23–24.5 μm long, 20.8–24 μm wide, 3–4 μm wide at the base, with five corners	***C. elsikii***

#### Catenularia angulospora

Taxon classificationFungiChaetosphaerialesChaetosphaeriaceae

(Linder) E.W. Mason, Mycol. Pap. 5: 121. 1941.

3C70D6BD-6754-5BB8-BCCF-987233CF5995

Fig. 3

Basionym. Haplochalara angulospora Linder, Mycologia 25: 347. 1933.
Synonym. ? Catenularia hughesii N.D. Sharma, J. Indian bot. Soc. 59: 73. 1980.

##### Description.

Colonies on natural substrate effuse, hairy to velutinous, dark brown to almost black. ***Anamorph*.** Conidiophores 77–220 × 4.5–6(–7) μm wide, 7–8 μm above the base, macronematous, solitary or arise in tufts, erect, straight or slightly flexuous, unbranched, dark brown, paler towards the apex, septate. Capitate hyphae absent. Conidiogenous cells 18–25 × 3.5–4.5 μm tapering to ca. 2.5 μm, integrated, terminal, monophialidic, extending percurrently, obclavate to subcylindrical or slightly lageniform, pale brown, paler towards the apex; collarettes 3–4 μm wide, 1.5(–2) μm deep, funnel-shaped, subhyaline, smooth, margin entire. Conidia 6–8(–9) μm long, 4.5–6(–7) μm wide at the apical end, ca. 2 μm wide at the basal hilum (mean ± SD = 7.4 ± 1.1 × 6.0 ± 1.2 μm × 2.0 ± 0.0 μm), rounded-obconic in side view, with three blunt corners when viewed from above, broadly rounded to flattened at the apex, truncate at the basal scar, pale brown to pale fuscous, thick-walled, smooth; formed singly, adhered in basipetal chains. ***Teleomorph*.** Unknown.

**Figure 3.**
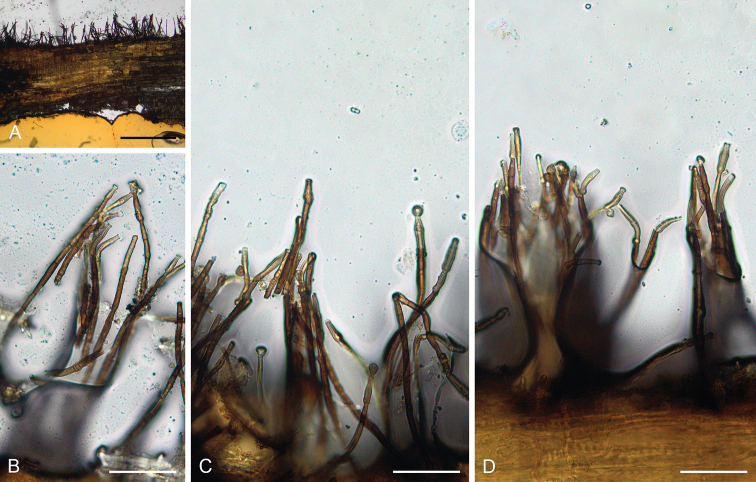
*Catenularia angulospora* (holotype FH 00965375) **A** vertical section of the wood with colony **B–D** conidiophores with conidia. Scale bars: 500 μm (**A**); 50 μm (**B–D**).

##### Specimen examined.

USA – Kentucky • near Louisville; on decaying beech log; 23 Mar. 1928; D.H. Linder (***holotype*** of *C. angulospora*FH herbarium 00965375, as microscopic slides).

##### Habitat and geographical distribution.

Saprobe on dead culms of *Bambusa* sp., decaying wood of *Fagus* sp. and other unknown hosts in freshwater and terrestrial habitats. It is known in China, India and the USA (Linder 1933; Sharma 1980; Luo et al. 2019 as *C. cubensis*).

##### Notes.

For additional description and illustration, see Luo et al. (2019, as *C. cubensis*). Hughes (1965) revised the type material of *H. angulospora*, and despite the striking similarities to other *Catenularia*, he kept the species in *Haplochalara* due to the absence of capitate hyphae. Sharma (1980) described *C. hughesii* on dead bamboo culms in India with pale brown to brown conidia 6–8 × 4.5–5.8 μm and conidiophores up to 270 × 5–7 μm. Although the holotype of this species was not available for study, a detailed morphological comparison of its original description and illustration with *C. angulospora* suggests that they are conspecific. Luo et al. (2019) reported this species as *C. cubensis* (strain MFLUCC 18-1331) from China, characterised by the absence of capitate hyphae and cuneiform, greyish-brown to brown conidia 6–8 × 4–6 μm.

In the phylogenetic analysis, the strain of *C. angulospora* MFLUCC 18-1331 clustered as a sister to *C. cubensis* S.M.H. 3258, but their relationship is not statistically supported. Both species are, however, very similar. *Catenularia cubensis* (Holubová-Jechová 1982) differs from *C. angulospora* in brown to dark brown conidia, slightly narrower at the apical end (5.5–8.5 × 3.5–5.5 μm), and presence of capitate hyphae scattered among the conidiophores. The ITS sequence identity between *C. cubensis* and *C. angulospora* is 96.5% and supports our conclusion to treat them as separate species.

#### Catenularia catenulata

Taxon classificationFungiChaetosphaerialesChaetosphaeriaceae

(Z.L. Luo, K.D. Hyde & H.Y. Su) Réblová & A.N. Mill. comb. nov.

0A5E677A-D408-5376-ABF9-9AAE6A68A26C

839462

Basionym. Chaetosphaeria catenulata Z.L. Luo, K.D. Hyde & H.Y. Su, Fungal Divers. 99: 582. 2019.

##### Habitat and geographical distribution.

Saprobe on submerged wood, known only in China (Luo et al. 2019).

##### Notes.

*Catenularia catenulata* is characterised by solitary conidiophores, absence of capitate hyphae and conidia 13–15 × 12–14 μm, greyish-brown, turbinate, triangular in side view with 3–4 corners when viewed from above (Luo et al. 2019). It resembles *C. malabrica* (Subramanian and Bhat 1989), but the latter species has larger conidia 12–18 × 18–21 μm with 4–5 corners.

#### Catenularia cubensis

Taxon classificationFungiChaetosphaerialesChaetosphaeriaceae

Hol.-Jech., Mycotaxon 15: 278. 1982.

9C9CE4E4-9054-51E4-B1D9-CF1A269DB513

Fig. 4

Synonym. Chaetosphaeria cubensis Hol.-Jech., Mycotaxon 15: 278. 1982.

##### Description.

Colonies on natural substrate effuse, hairy to velutinous, dark brown, mycelium partly immersed, partly superficial, brown; colonies composed of conidiophores, capitate hyphae and sometimes ascomata. ***Anamorph*.** Conidiophores 115–200 × 4–4.5 μm, 4.5–6 μm wide above the base, macronematous, solitary or arise in tufts, erect, straight or flexuous, unbranched, thick-walled, brown to dark brown, slightly paler towards the apex. Capitate hyphae 104–165 × 4–4.5 μm, 4–5.5 μm wide above the base, arise among the conidiophores, extending percurrently, erect, straight, brown to dark brown, paler towards the apex, apical cell sterile, thin-walled, subhyaline, slightly swollen, ca. 3.5 μm wide, broadly rounded, the hyaline gelatinous cap was not observed. Conidiogenous cells 22–38 × 3.5–4.5 μm tapering to 2–2.5 μm below the collarette, terminal, integrated, monophialidic, extending percurrently, cylindrical, pale brown to brown, producing conidia successively; collarettes 3.5–4 μm wide, 1–2 μm deep, shallow, funnel-shaped, pale brown, smooth, margin entire. Conidia 5.5–8.5 μm long, 3.5–5.5 μm wide at the apical end, 1.5–2 μm wide at the basal hilum (mean ± SD = 7.5 ± 0.7 × 4.3 ± 0.4 × 1.8 ± 0.2 μm), rounded-obconic to broadly obovoid in side view, with three bluntly rounded corners when viewed from above, broadly rounded to flattened at the apex, truncate at the basal scar, aseptate, brown to dark brown, thick-walled, smooth; formed singly, adhered in basipetal chains. ***Teleomorph*.** Ascomata 150–200 µm diam, 160–210 µm high, superficial, solitary or in groups, subglobose to conical, papillate, dark brown to black, covered with conidiophores and capitate hyphae. Ostiole periphysate. Ascomatal wall fragile, carbonaceous, 15–25 μm thick, two-layered. Outer layer consisting of brown, polyhedral cells with opaque walls. Inner layer consisting of several rows of thin-walled, hyaline cells. Paraphyses 2.5–3.5 μm wide, septate, hyaline, longer than the asci. Asci 62–84.5 × (6–)7–8.5 μm (mean ± SD = 72.2 ± 7.8 × 13.9 ± 0.9 µm), cylindrical-clavate, short-stipitate, apically rounded to obtuse, ascal apex with a non-amyloid apical annulus 2–2.5 μm wide, 1.5(–2) μm high. Ascospores 12–16(–17.5) × 3–4 μm (mean ± SD = 13.9 ± 0.9 × 3.5 ± 0.2 µm), fusiform, straight or slightly curved, hyaline, 3-septate, smooth, 2-seriate in the ascus.

**Figure 4.**
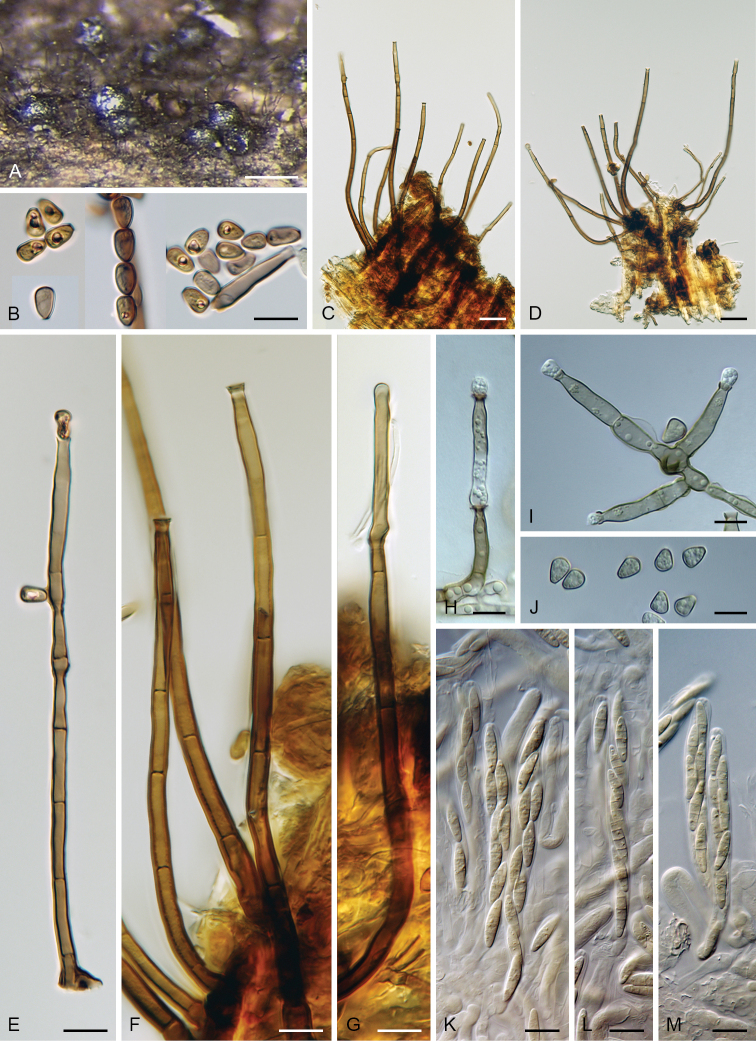
*Catenularia cubensis***A** ascomata accompanied by conidiophores and capitate hyphae **B, J** conidia **C, D** tufts of conidiophores with scattered capitate hyphae **E–I** conidiophores **K–M** asci with ascospores. Images: S.M.H. 3258 (**A, H–J**), PRM 825347 holotype (**B–D, F, G, K–M**); PRA-19884 (**E**); on natural substrate (**A–G, K–M**); in culture (**H–J**). Scale bars: 200 μm (**A**); 10 μm (**B, E–M**); 25 μm (**C, D**).

##### Specimens examined.

Costa Rica • Guanacaste, Liberia ACG, Sector Santa Maria, Estacion Biologica, trail to Bosque Encantado; 10.7647N, -85.3033W; alt. 750 m; on 5 cm diam branch on ground; 26 Jun. 1997; S.M. Huhndorf (S.M.H. 3258). Costa Rica • Alajuela, Cantón Upala, District Bijagua, Heliconias Station, Heliconias trail; 10.7081N, -85.0453W; on 25 cm diam log on ground; alt. 1190 m; 12 Jul. 2001; S.M. Huhndorf, F.A. Fernández, A.N. Miller & M. Darin (S.M.H. 4454). Cuba – Isla de la Juventud (Isla de Pinos) • Sierra de Casas, in a valley near El Abra, 2 km SW of Nueva Gerona; on dead trunk of Palmaceae; 22 Jan. 1981; V. Holubová-Jechová (***holotype***PRM 825347). Cuba – Isla de la Juventud (Isla de Pinos) • in forest near village Caryo Piedra; on wood of a trunk of a deciduous tree; 21 Jan. 1981; V. Holubová-Jechová (PRA-19884).

##### Habitat and geographical distribution.

Saprobe on decaying wood of palm *Euterpe oleracea* and other hosts in Brazil, Cuba and Costa Rica (Holubová-Jechová 1982; De Castro et al. 2011; Miller and Huhndorf, unpubl.; this study), and on fallen leaves in India (Dubey and Pandey 2017).

##### Notes.

The description is based on Cuban collections. In the Costa Rican material, conidia were 6–8.5 μm long, 3–5 µm wide at the widest point, 1.5–2 µm wide at the basal hilum, brown to dark brown, broadly obovoid or cuneiform, asci 60–80 × 7–9 µm, ascospores 12–20 × 3–5 µm, fusiform, 3-septate (Huhndorf and Miller, unpubl.). For additional details, see Holubová-Jechová (1982).

*Catenularia cubensis* closely resembles *C. angulospora*; for comparison see notes for the latter species. *Catenularia minor* can also be compared with *C. cubensis*, but differs in longer and wider conidia (6.5–)7.5–10.5(–13) × 6.5–11.5 μm with 3–5 blunt corners and conidiophores that form two distinct layers.

#### Catenularia cupulifera

Taxon classificationFungiChaetosphaerialesChaetosphaeriaceae

(Berk. & Broome) Réblová & A.N. Mill. comb. nov.

4E9C1741-9265-5080-8FFB-2C716B765A58

839463

Fig. 5

Basionym. Sphaeria cupulifera Berk. & Broome, Ann. Mag. nat. Hist., Ser. 4, 7: 435. 1871.
Synonyms. Lasiosphaeria cupulifera (Berk. & Broome) Cooke & Plowr., Grevillea 7(43): 85. 1879.
Chaetosphaeria cupulifera (Berk. & Broome) Sacc., Syll. fung. 2: 94. 1883.
Psilonia cuneiformis Richon, Bull. Soc. Sci. Vitry-le-Franç. 8: 219. 1877.
Monotospora cuneiformis (Richon) Sacc., Syll. fung. 4: 300. 1886.
Psiloniella cuneiformis (Richon) Costantin, Mucéd. Simpl.: 86. 1888.
Catenularia cuneiformis (Richon) E.W. Mason, Mycol. Pap. 5: 121. 1941.
Catenularia simplex Grove, Syll. fung. 4: 303. 1886.
Psilonia simplex (Grove) Costantin, Mucéd. Simpl.: 86. 1888.
Synonymy adopted from Mason (1971) and Booth (1958).

##### Description.

Colonies on natural substrate effuse, hairy or tufted, dark brown to black, mycelium partly immersed, partly superficial, brown; colonies composed of conidiophores, capitate hyphae and sometimes ascomata. ***Anamorph*.** Conidiophores 100–350(–530) × 6–7.5(–8) μm, 8.5–10.5 wide above the base, macronematous, solitary or in tufts, with dark brown stromatic hyphal cells around the bases, erect, straight or flexuous, unbranched, brown to dark brown, thick-walled, slightly paler towards the apex. Capitate hyphae 110–160 × 5.5–6 μm, 6.5–7 μm wide above the base, scattered among the conidiophores, erect, straight, brown to dark brown, paler towards the apex, apical cell sterile, thin-walled, subhyaline, slightly swollen, ca. 7 μm wide, broadly rounded with a hyaline gelatinous cap that disappears with age. Conidiogenous cells 40–59 × 5.5–6.5 μm, not tapering, terminal, integrated, monophialidic, extending percurrently, cylindrical, brown, producing conidia successively; collarettes 9.5–12.5 μm wide and 10–12.5 μm deep, funnel-shaped, brown, slightly roughened, with an irregularly frayed margin. Conidia (9–)10.5–13.5 μm long, 7–9.5 μm wide at the apical end, 3.5–4.5 μm wide at the basal hilum (mean ± SD = 11.8 ± 0.7 × 8.0 ± 0.6 μm × 4.0 ± 0.3 μm), cuneiform in side view, with (3–)4(–5) blunt corners when viewed from above, flattened to broadly rounded at the apex, truncate at the base, aseptate, fulvous, brown to dark brown, thick-walled, smooth; formed singly, adhered in basipetal chains.

**Figure 5.**
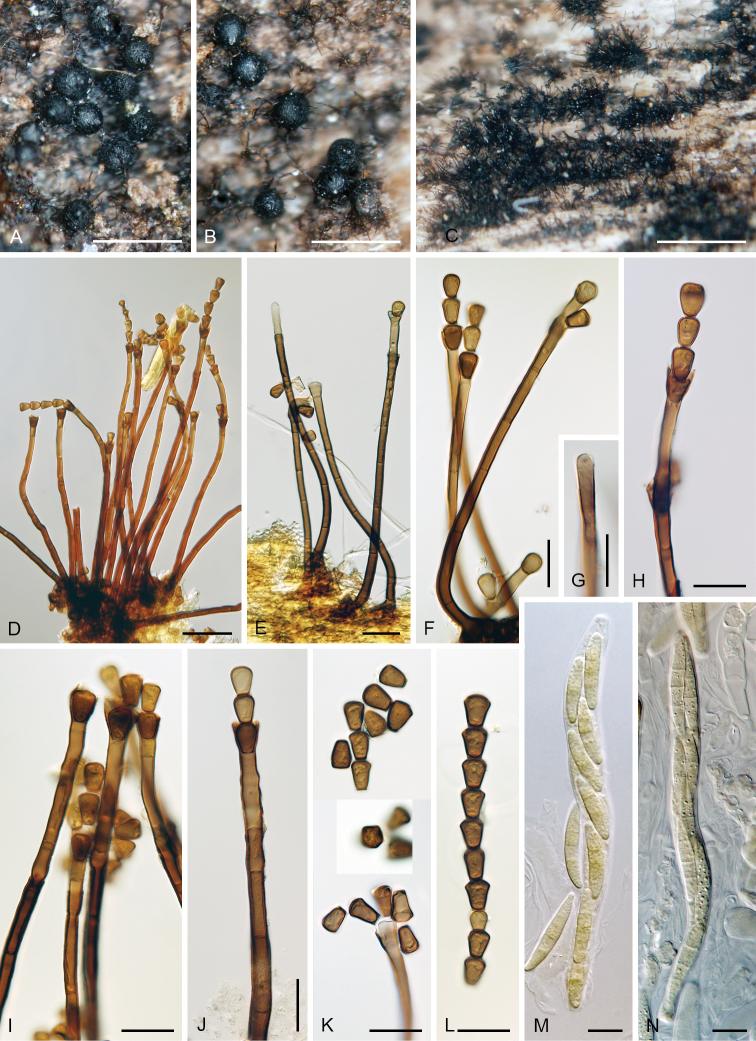
*Catenularia cupulifera***A, B** ascomata accompanied by conidiophores and capitate hyphae **C** colony composed of conidiophores and capitate hyphae **D–F** conidiophores **G** capitate hypha **H–J** upper parts of conidiophores with conidia **K, L** conidia **M, N** asci with ascospores. Images: W7972 (**A, B, M**); W7973 (**C, D, H, I**); PRA-19893 (**E–G, J–L**); JF 99018 (**N**); on natural substrate (**A–N**). Scale bars: 500 μm (**A–C**); 50 μm (**D**); 25 (**E**); 20 μm (**F–L**); 10 μm (**M, N**).

##### *Teleomorph*.

Ascomata 150–220 μm diam, 200–250 μm high, superficial with a base immersed, solitary or in groups or densely aggregated forming a crust, conical to subglobose, papillate, dark brown to black, rugose, sometimes covered with conidiophores and capitate hyphae or in a dense subiculum consisting of partly decumbent conidiophores. Ostiole periphysate. Ascomatal wall fragile, carbonaceous, 22–33 μm thick, two-layered. Outer layer consisting of brown, polyhedral to angular cells with opaque walls. Inner layer consisting of several rows of thin-walled, hyaline cells. Paraphyses 3–4 μm wide tapering to 2–2.5 μm, septate, hyaline, longer than the asci. Asci 110–140 × (8–)10–11(–12.5) μm (mean ± SD = 162.2 ± 11.1 × 10.5 ± 1.2 µm), cylindrical-clavate, short-stipitate, apically narrowly rounded to obtuse, ascal apex with a non-amyloid apical annulus 2–2.5(–3) μm wide, ca. 1.5 μm high. Ascospores 21–28.5 × 4.5–5.5 μm (mean ± SD = 25.3 ± 1.7 × 5.5 ± 0.4 µm), fusiform, straight or slightly curved, hyaline, 1–4-septate, smooth, 2-seriate in the ascus.

##### Specimens examined.

Belgium • West Flanders province, Adinkerke, Cabour; on decaying wood of *Populus* sp.; 21 Oct. 2007; B. Declerque (IFBL D0.16.23). Czech Republic – Moravia • Lanžhot, Ranšpurk National nature reserve; alt. 150 m; on decaying wood of *Carpinus betulus*; 14. Aug. 1979; V. Holubová-Jechová (PRA-19887) • *Ibid.*; on decaying wood of *Populus alba*, 28 Jul. 1970, V. Holubová-Jechová (PRA-19888) • *Ibid.*; on decaying wood *Quercus robur*, 28. Aug. 1976, V. Holubová-Jechová (PRA-19889). Czech Republic – Moravia • Bílé Karpaty, Velká Javořina Mt. near Kamenná Bouda; alt. 660 m; on decaying wood of a branch of *Fagus sylvatica*; 27 Jul. 1970; V. Holubová-Jechová (PRA-19886). France – Ariège • Pyreneés Mts., Rimont, Las Muros, alt. 480 m; on decaying wood of *Fraxinus excelsior*; 4 Feb. 1999; J. Fournier J.F. 99018 (PRA-19890). France – Ariège • Pyreneés Mts., Rimont, Las Muros, valley of the Peyrau brook, alt. 400 m; on decaying wood of *Buxus sempervivens*; 9 Nov. 1999, J. Fournier J.F. 99261 (PRA-19892) • *Ibid.*; on decaying wood of *Salix caprea*; 12 Mar. 2000; J. Fournier J.F. 00026 (PRA-19891). France – Ariège • Pyrénées Mts., Rimont, La Maille brook; alt. 550 m; on submerged wood; 28 May 2018; J. Fournier M.R. 4104 (PRA-19893). Slovak Republic • Brezová near Senica; on decaying wood of a trunk of *Salix alba*; 6 Aug. 1976; V. Holubová-Jechová (PRA-19885). United Kingdom – Somerset • Langridge, on decaying wood of roots of *Ulmus* sp.; Apr. 1869; C.E. Broome (***holotype*** of *S. cupulifera* K(M) 57177). United Kingdom • on decaying wood; 14 Apr. 1873; ex Herbarium C.E. Broome 1886 (W 7972) • *Ibid.*; ex Herbarium C.E. Broome 1886, no. 366 (W 7973).

##### Habitat and geographical distribution.

Saprobe on decaying wood of *Carpinus betulus*, *Fagus sylvatica*, *Fraxinus excelsior*, *Hedera* sp., *Ilex* sp., *Quercus* sp., *Salix alba*, *Ulmus* sp. and other unknown hosts. Most of the records originate from Europe in Belgium, Czech Republic, France, Slovak Republic and the United Kingdom (Berkeley and Broome 1871; Hughes 1965; Holubová-Jechová 1973; this study). Hughes (1965) suggested that *C. cupulifera* is apparently only known from Europe. However, findings of this species also come from other continents. *Catenularia cupulifera* has been reported from foam in a river in Venezuela (Fernández and Smits 2018), wood of *Ulmus americana* in the USA, Illinois (Shim 1969) and decaying leaves of *Pandanus* sp. in Mauritius (Whitton et al. 2012).

##### Notes.

Our observations of the teleomorph-anamorph connection between *Ch. cupulifera* and *C. cuneiformis* agree with those of Berkeley and Broome (1871), De Seynes (1886) and Booth (1958). Although this relationship has not yet been verified experimentally, both morphs occur together in nature. Since the anamorph and teleomorph represent two different stages of the life cycle of one organism, we propose a new combination in *Catenularia* based on *Sphaeria cupulifera* with *C. cuneiformis* and *C. simplex* as synonyms.

*Catenularia novae-zelandiae* resembles *C. cupulifera* but differs in larger and rounded-obconic conidia, 11.5–17.5 μm long, 14.5–18.5 μm wide. Both species have conspicuous collarettes with a frayed margin, which is larger in *C. novae-zelandiae*, 19–27 μm wide and 12.2–19 μm deep, funnel- to cup-shaped.

#### Catenularia elsikii

Taxon classificationFungiChaetosphaerialesChaetosphaeriaceae

(M.J. Pound, J.M.K. O’Keefe, N.B. Nuñez Otaño & J.B. Riding) Réblová & A.N. Mill. comb. nov.

73255A35-8D2C-58EA-94B3-685B8A12EB0B

839464

Basionym. Chaetosphaeria elsikii M.J. Pound, J.M.K. O’Keefe, N.B. Nuñez Otaño & J.B. Riding, Palynology 43: 603. 2019.

##### Habitat and geographical distribution.

On fossil wood, known only in the United Kingdom.

##### Notes.

*Catenularia elsikii* was isolated from the material containing clay, charcoal and wood fragments present in the cracks of a large sample of fossil wood discovered in the United Kingdom (Pound et al. 2019). Thick-walled, dark brown conidia were the only structure that has been preserved in material dated to the Miocene. In the conidial characteristics, *C. elsikii* is remarkably similar to *C. macrospora* known from Canada and New Zealand and *C. novae-zelandiae* known only from New Zealand. These species share dark brown, rounded-obconic conidia with (3–)4–5 corners when viewed from above. In addition, *C. elsikii* and *C. novae-zelandiae* have a visible pore at the basal hilum. Conidia of *C. elsikii* (23.1–24.4 μm high, 20.8–23.9 μm wide with a basal scar 3–4 μm wide) are longer and wider than those of *C. novae-zelandiae*, but shorter than those of *C. macrospora*. For detailed comparison, see notes to the two latter species.

#### Catenularia kalakadensis

Taxon classificationFungiChaetosphaerialesChaetosphaeriaceae

Subram. & Bhat, Kavaka 15(1–2): 49. 1989 [1987].

44CF6C23-E3CF-57DB-85B5-32C608F33BC2

##### Habitat and geographical distribution.

Saprobe on decaying wood, known only in China, India and Mexico (Subramanian and Bhat 1989; Heredia et al. 2004; Xia et al. 2013).

##### Notes.

For descriptions and illustrations, refer to Subramanian and Bhat (1989) and Xia et al. (2013). *Catenularia kalakadensis* is unique among other species in conidia with six blunt corners when viewed from above. It resembles *C. cubensis* but differs in the absence of capitate hyphae and wider conidia (6–7 μm) with more corners at the apex (Subramanian and Bhat 1989).

#### Catenularia longispora

Taxon classificationFungiChaetosphaerialesChaetosphaeriaceae

S. Hughes, N. Z. J. Bot. 3: 141. 1965.

B719586D-0913-5779-8831-8F8D0A555EAD

##### Habitat and geographical distribution.

Saprobe on decaying wood, known only in New Zealand (Hughes 1965).

##### Notes.

*Catenularia longispora* is well recognisable by narrowly rounded-obconic, brown to dark brown conidia that are the longest in the genus, 27–45 μm long, 16.8–24 μm wide at the apical end, 7–10 μm wide at the basal hilum, with usually three blunt corners when viewed from above (Hughes 1965).

#### Catenularia macrospora

Taxon classificationFungiChaetosphaerialesChaetosphaeriaceae

S. Hughes, N. Z. J. Bot. 3: 143. 1965.

3E3D8A16-7603-5F3F-B121-76DEDB5F2A6E

##### Habitat and geographical distribution.

Saprobe on decaying bark and wood of *Dacrydium cupressinum*, *Fuscospora cliffortioides*, *Vitex lucens* and other unknown hosts, known in Canada and New Zealand (Hughes 1965).

##### Notes.

*Catenularia macrospora* has broadly obovoid to rounded-obconic, brown to dark brown conidia, 21–28 μm long, 19–28 μm wide at the apical end and 4–7 μm wide at the basal hilum, with (3–)4(–5) blunt corners when seen from above (Hughes 1965). The conidial length is comparable with those of *C. longispora* and *C. elsikii*, but the former species differs in conidia narrowly rounded-obconic, narrower at the apical end (16.8–24 μm) with only (2–)3 corners. Although the length of conidia of *C. elsikii* and *C. macrospora* overlap and the number of corners is comparable, conidia of *C. elsikii* are slightly shorter and narrower in their upper range (23–24.5 × 21–24 μm) and narrower at the truncate base (3–4 μm) (Pound et al. 2019).

#### Catenularia malabarica

Taxon classificationFungiChaetosphaerialesChaetosphaeriaceae

Subram. & Bhat, Kavaka 15(1–2): 49. (1989) [1987].

39AD616C-B64C-569F-B928-9C306F60F3BC

##### Habitat and geographical distribution.

Saprobe on decaying wood of *Magnolia liliifera* and an unknown host, known only in India and Thailand (Subramanian and Bhat 1989; Kodsueb et al. 2008).

##### Notes.

For descriptions and illustrations, see Subramanian and Bhat (1989). *Catenularia malabrica* produces one of the tallest conidiophores in the genus, 320–620 × 6–11 μm arising singly or in tufts. It resembles *C. novae-zelandiae* in dark brown conidia with 4–5 corners, but conidia of *C. malabrica* are wider (18–21 μm) and the capitate hyphae are absent.

#### Catenularia minor

Taxon classificationFungiChaetosphaerialesChaetosphaeriaceae

(Hol.-Jech.) Réblová & A.N. Mill. comb. nov.

6A1E433B-6EEE-55D4-A7B5-FBDE41833AF3

839465

Fig. 6

Basionym. Catenularia cuneiformis var. minor Hol.-Jech., Česká Mykol. 37: 14. 1983.
Synonym. Chaetosphaeria trianguloconidia Réblová & Seifert, Sydowia 55: 333. 2003.

##### Description.

Colonies on the natural substrate effuse, tufted or hairy, dark brown to black, mycelium partly immersed, partly semi-immersed, pale brown to brown; colonies composed of conidiophores, capitate hyphae and sometimes ascomata. ***Anamorph*.** Conidiophores macronematous, solitary or arise in tufts, with dark brown stromatic hyphal cells around the base, erect, straight or flexuous, unbranched, thick-walled, paler towards the apex, forming two layers. Conidiophores of the lower layer 95–212 × 3.5–4.5(–5) μm, 4.5–5.5(–8.5) μm wide above the base, pale brown to brown; conidiophores of the upper layer 260–527 × 4.5–7 μm long, 7.5–10 μm wide above the base, dark brown. Capitate hyphae 122–186 × 3.5–5 μm, 5–5.5 μm wide above the base, scattered among the conidiophores, erect, straight, brown, extending percurrently, paler towards the apex, apical cell subhyaline, slightly swollen, 3.5–4 μm wide, broadly rounded, thin-walled; the hyaline gelatinous cap was not observed. Conidiogenous cells 15–40 × 3.5–5.5 μm tapering to 2.5–3 μm below the collarette, integrated, terminal, monophialidic, extending percurrently, cylindrical to slightly lageniform, pale brown to brown, producing conidia successively; collarettes 3.5–5(–6) μm wide, 1.5–2.5 μm deep, shallow, funnel-shaped, pale brown to subhyaline, smooth, margin entire. Conidia (6.5–)7.5–10.5(–13) μm long, 6.5–11.5 μ wide at the apical end, 1.5–2.5 μm wide at the base (mean ± SD = 8.9 ± 0.9 × 9.0 ± 1.2 × 2.1 ± 0.2 μm), cuneiform to rounded-obconic to obtriangular in side view, with 3–5 blunt corners when viewed from above, broadly rounded to flattened at the apex, truncate at the basal scar with a central pore, aseptate, pale brown to dark brown, thick-walled, smooth; formed singly, adhered in basipetal chains or clusters. ***Teleomorph*.** Ascomata 230–250 μm diam, 250–275 μm high, superficial, solitary or densely aggregated, subglobose to globose, covered by a whitish-grey powder except for the black glabrous papilla; the powdery covering is ca. 5–15 μm thick, disappearing with age, leaving the perithecia dark and glabrous. Ascomata sparsely covered with conidiophores. Ostiole periphysate. Ascomatal wall fragile, carbonaceous, 30–37.5 μm thick, two-layered. Outer layer consisting of dark brown, opaque, thin-walled, polyhedral cells. Inner layer consisting of hyaline, thinner-walled, elongated, compressed cells. Paraphyses 3–4 μm wide, tapering to ca. 2 μm, branching, anastomosing, septate, hyaline, longer than asci. Asci 102–112 × 8–9(–9.5) μm (mean ± SD = 106 ± 1.6 × 8.9 ± 0.2 µm), cylindrical-clavate, short-stipitate, rounded apically, ascal apex with a non-amyloid apical annulus 3 μm diam, 1.5–2 μm high. Ascospores 25–29(–30) × (3.5–)4–4.5 μm (mean ± SD = 27 ± 0.5 × 4 ± 0.7 µm), fusiform, straight or curved, hyaline, 1–3-septate, smooth, 1–2-seriate in the ascus (adapted from Réblová and Seifert 2003).

**Figure 6.**
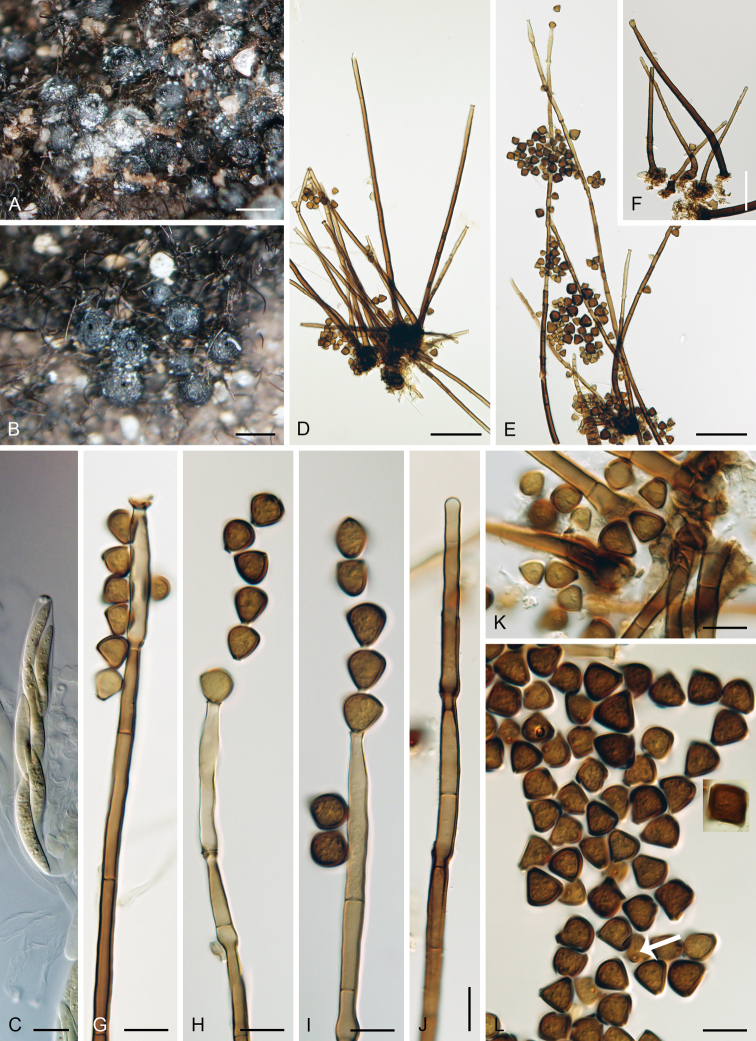
*Catenularia minor***A, B** colonies composed of ascomata, conidiophores and capitate hyphae **C** ascus with ascospores **D–F** conidiophores with capitate hyphae **G–J** upper parts of conidiophores with conidia in chains **J** capitate hypha **K, L** conidia (arrow indicates central pore in the basal scar). Images: PRM 828704 holotype of *C. minor* (**D, E, G, K**); PRM 900544 holotype of *Ch. trianguloconidia* (**A–C, F, H–J, L**); on natural substrate (**A–L**). Scale bars: 250 μm (**A, B**); 10 μm (**C, G–L**); 50 μm (**D–F**).

##### Specimens examined.

Cuba – Sancti Spiritus province • Soledad, Cienfuegos Province Botanical Garden; on decaying stem of *Bambusa vulgaris*; 19 Mar. 1981; M.A. Bondarceva & S. Herrera (***holotype*** of C. cuneiformis var. minorPRM 828704). Thailand – Nakhon Nayok Province • Khao Yai National Park, trail to Haew Suwat waterfall, elev. 720 m; on decaying bamboo culm; 2 Sep. 2001; M. Réblová, Gary J. Samuels & R. Nasit M.R. 2186/TH 438 (***holotype*** of *Ch. trianguloconidia*PRM 900544).

##### Habitat and geographical distribution.

Saprobe on dead culms of bamboo, known in Cuba and Thailand (Holubová-Jechová 1983; Réblová and Seifert 2003).

##### Notes.

For characteristics in culture, see Réblová and Seifert (2003). The apparent similarity of C. cuneiformis var. minor (Holubová-Jechová 1983) and *Ch. trianguloconidia* (Réblová and Seifert 2003) and their habitat on dead bamboo culms prompted a revision of both species. Examination of their holotypes revealed that they are conspecific. Additionally, we discovered capitate hyphae in the type material of both species, although they were not described in the protologues. They are scattered among the conidiophores and easy to overlook. The hyaline gelatinous cap around the swollen apex of the capitate hyphae was not observed. Conidia slightly vary in size and colour, and often smaller and pale brown conidia occur together with slightly larger and darker brown conidia.

Holubová-Jechová (1983) distinguished var. minor from var. cuneiformis (= *C. cupulifera*, this study) in shorter collarettes, smaller conidia and the absence of capitate hyphae. Based on their different morphology, a new combination for var. minor is proposed at the species level with *Ch. trianguloconidia* reduced to synonymy.

*Catenularia angulospora* is similar to *C. minor*, and it is challenging to distinguish both species, especially if capitate hyphae may rarely occur in some specimens of the latter species. *Catenularia angulospora* differs in fuscous to brown conidia that are narrower (4.5–6(–7)) μm and the lack of capitate hyphae. *Catenularia cupulifera* is comparable to *C. minor* but differs in larger collarettes (9.5–12.5 μm wide and 10–12.5 μm deep) with a frayed margin, and longer (10.5–13.5 μm) conidia that are wider (3.5–4.5 μm) at the basal hilum. Conidia of *C. cupulifera* are cuneiform in side view, whereas conidia of *C. minor* are more rounded-obconic to obtriangular.

#### Catenularia novae-zelandiae

Taxon classificationFungiChaetosphaerialesChaetosphaeriaceae

(S. Hughes & Shoemaker) Réblová & A.N. Mill. comb. nov.

A515EA17-4A3B-57D5-8FD5-6C6A08E887DD

839466

Fig. 7

Basionym. Chaetosphaeria novae-zelandiae S. Hughes & Shoemaker, N. Z. J. Bot. 3: 138. 1965.

##### Description.

Colonies on natural substrate effuse, tufted or velutinous, dark brown, mycelium partly immersed, partly superficial, brown; colonies composed of conidiophores, capitate hyphae and sometimes ascomata. ***Anamorph*.** Conidiophores 90–354 × 7.5–9.5 μm, 7–10.5 μm wide near the swollen base, macronematous, solitary or arise in tufts, with dark stromatic hyphal cells around the base, erect, straight or flexuous, unbranched, brown to dark brown, thick-walled. Capitate hyphae 95–215 × 5–7 μm, 6.5–9 μm wide above the base, 4.5–5.5 μm wide at the apex, solitary or in tufts, arise among the conidiophores, erect, straight to slightly flexuous, dark brown, paler towards the apex, apical cell pale brown to subhyaline, slightly swollen, broadly rounded, thin-walled, with a hyaline, mucilaginous cap that disintegrates with age. Conidiogenous cells 22.5–41(–65) × 7–11 μm, 7.5–9.5 μm wide below the collarette, terminal, integrated, monophialidic, extending percurrently, cylindrical, subcylindrical or slightly lageniform, brown, producing conidia successively; collarettes 19–27 μm wide and 12.2–19 μm deep, funnel-shaped or cup-shaped, brown to dark brown, roughened, with a frayed margin, the margin deteriorates, and the collarette becomes reduced in size 11.5–15.8 μm wide and 4.5–6 μm deep. Conidia 11.5–17.5 μm long, 14.5–18.5 μm wide at the apical end, 4–5.5 μm wide at the basal hilum, (mean ± SD = 15.8 ± 1.8 × 15.9 ± 1.3 × 5.5 ± 0.9 μm), cuneiform to rounded-obconic in side view, with 4–5 blunt corners when viewed from above, flattened to broadly rounded at the apex, truncate at the base, aseptate, brown to dark brown, thick-walled, smooth; formed singly, adhered in basipetal chains. ***Teleomorph*.** Ascomata 160–210 μm diam, 180–220 μm high, superficial, solitary or in small groups, subglobose to globose, papillate, dark brown, sometimes covered with capitate hyphae and conidiophores; capitate hyphae 80–130 × 5–5.5 μm, erect, simple, apical cell 6–6.5 μm wide, slightly inflated, broadly rounded apically, subhyaline, with a mucilaginous cap that disappears with age. Ostiole periphysate. Ascomatal wall fragile, carbonaceous, 17–22 μm thick, two-layered. Outer layer consisting of dark brown, polyhedral to angular cells with opaque walls. Inner layer consisting of rows of thin-walled, hyaline cells. Paraphyses 4–5 μm wide tapering to 1.5–2 μm, septate, hyaline, longer than the asci. Asci 102–130 × 11–13 μm (mean ± SD = 117.6 ± 9.8 × 12.3 ± 0.8 µm), 74–100(–110) μm in the sporiferous part (mean ± SD = 83.7 ± 12 µm), cylindrical-clavate, narrowly truncate apically, ascal apex with a non-amyloid apical annulus 3.5–4 μm wide, ca. 2 μm high. Ascospores 22–28(–30) × 4–5 μm (mean ± SD = 25.6 ± 1.6 × 4.7 ± 0.4 µm), fusiform, straight or slightly curved, hyaline, 3-septate, smooth, 2-seriate in the ascus.

**Figure 7.**
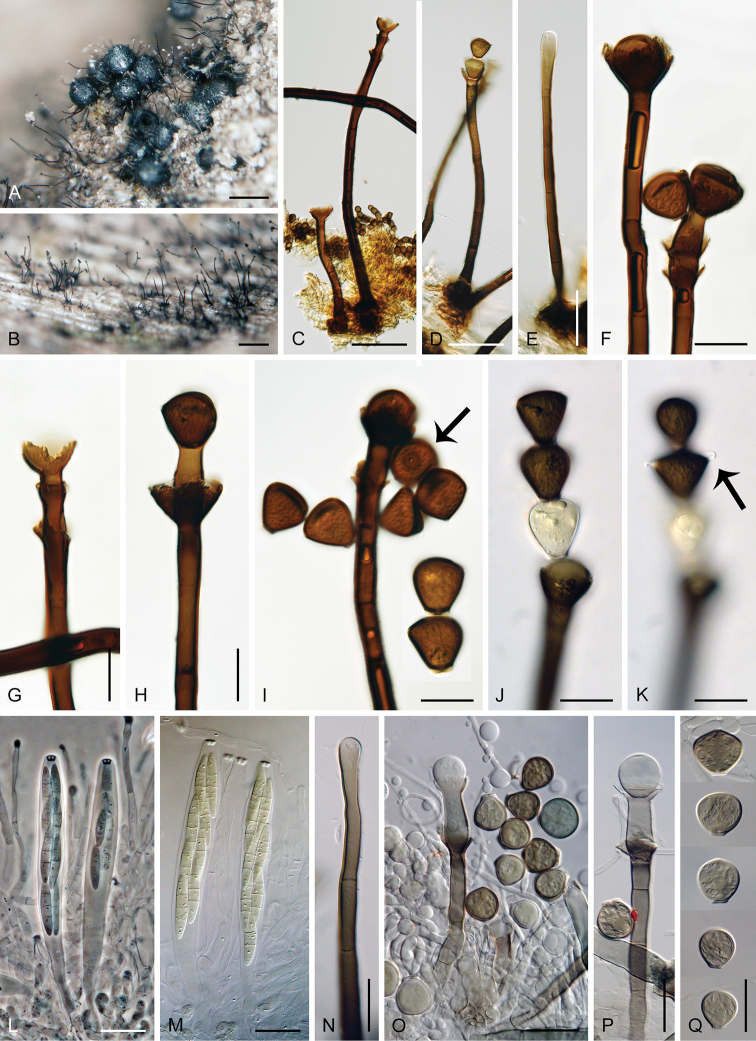
*Catenularia novae-zelandiae***A** ascomata **B** colony composed of conidiophores and capitate hyphae **C, D, O, P** conidiophores **E** capitate hypha arising among conidiophores **F–I** upper parts of conidiophores with conidia (arrow indicates central pore at the basal scar) **J, K** conidia in chain (arrow indicates appendages) **L, M** asci with ascospores and paraphyses **N** capitate hypha arising from the ascomal wall **Q** conidia. Images: PDD 81883 (**A–C, F–I, L–Q**); PDD 119362 (**D, E, J, K**); on natural substrate (**A–N**); in PCA culture after 2 wk (**O–Q**). Scale bars: 250 μm (**A, B**); 50 μm (**C–E**); 20 μm (**F–Q**).

##### Characteristics in culture.

On PCA: colonies 8–12 mm in 14d, circular, flat, margin entire, subsurface, aerial mycelium scarce, cobwebby to mucoid, beige-brown, reverse of the same colour. Sporulation was abundant, sporulating conidiophores developed from aerial mycelium and occasionally from immersed vegetative hyphae.

Colonies on PCA effuse, hairy, vegetative mycelium subhyaline to hyaline, 2–3 μm wide. Conidiophores, conidiogenous cells and conidia similar to those from nature. Conidiophores 31–120 × 6–7 μm, solitary or arise in tufts of 2–7, erect, straight, pale brown, 1–several-septate. Capitate hyphae absent. Conidiogenous cells 22–37 × 8–10 μm, tapering to ca. 7 μm below the collarette; collarettes 12.5–15 μm wide, 4–6(–8) μm deep, funnel-shaped, pale brown to dark brown, slightly roughened with a frayed to entire margin. Conidia (13–)14–18 μm long, 13–18 μm wide at the apical end, 4.5–6 μm wide basal hilum (mean ± SD = 15.2 ± 1.2 ×14.7 ± 1.4 µm × 5.5 ± 0.9 µm), broadly rounded-obconic in side view, aseptate, brown to grey-brown, thick-walled, smooth, formed singly, adhered in short basipetal chains.

##### Specimens examined.

New Zealand – Auckland region • Auckland district, Upper Piha Valley, Waitākere Ranges, Home track; on decaying wood of *Metrosideros robusta*; 9 Oct. 1963; J.M. Dingley (***holotype***PDD 21603, ***isotype***DAOM 93575). New Zealand – West Coast region • Westland district, Otira, Kelly Shelter, Cockayane Nature Walk; on decaying wood; 16 Mar. 2003; M. Réblová MR 2846/NZ 362 (PDD 81883). New Zealand – West Coast region • Buller district, Victoria Forest Park, Reefton, Big River Inanganua track ca. 14 km; on decaying wood of *Nothofagus* sp.; 6 Mar. 2003; M. Réblová MR 2723/ NZ 224A (PDD 119362).

##### Habitat and geographical distribution.

Saprobe on decaying wood of *Coprosma lucida*, *Coprosma* spp., *Freycinetia banksii*, *Griselinia lucida*, *Leptospermum ericoides*, *Metrosideros robusta*, *Neopanax arboreum*, *Nothofagus* sp., *Olearia rani*, *Weinmannia racemosa* and other unknown hosts, known only in New Zealand (Hughes 1965; this study).

##### Notes.

The specimen PDD 81883 of *C. novae-zelandiae* was isolated in axenic culture (Fig. 7O–Q). In vitro, conidia were paler than those from nature and broadly rounded-obconic. Unfortunately, the isolate is no longer viable. The other collection PDD 119362 has conidia slightly larger 17.5–21 × 18–19 μm, 5–6 μm wide at the truncate base. In both specimens, we observed several conidia with minute hyaline appendages arising from the pale, circular, thin-walled areas in the cell wall (Fig. 7K).

*Catenularia malabarica* (Subramanian and Bhat 1989) is similar to *C. novae-zelandiae* in characters of conidia, but differs in the absence of capitate hyphae, longer conidiophores up to 620 μm long and conidiogenous cells with a shallow, funnel-shaped collarette without a frayed margin.

#### Chalarodes

Taxon classificationFungiChaetosphaerialesChaetosphaeriaceae

McKenzie, Mycotaxon 42: 89. 1991.

4482E560-9608-53C3-B1BC-6FE396075CB7

##### Description.

Colonies on natural substrate effuse, hairy, mycelium partly superficial, partly immersed; colonies composed of conidiophores and sometimes ascomata. ***Anamorph*.** Setae present, mostly associated with ascomata, simple, brown, apically rounded. Conidiophores mononematous, macronematous, solitary, erect, septate, unbranched, brown. Conidiogenous cells integrated, terminal, monophialidic, extending percurrently, cylindrical-lageniform to urceolate, brown; collarettes funnel-shaped, pale brown. Conidia obpyramidal, in side view cuneiform, obovoid to obtriangular, with angular outline when viewed from above, truncate at the basal scar, with a simple setula inserted apically at each corner, aseptate, hyaline, adhered in basipetal chains. ***Teleomorph*.** Ascomata non-stromatic, perithecial, papillate, dark brown, sparsely covered by setae and conidiophores. Ostiole periphysate. Ascomatal wall fragile, carbonaceous, two-layered. Paraphyses persistent, septate, hyaline, longer than the asci. Asci unitunicate, 8-spored, cylindrical-clavate, ascal apex with a non-amyloid apical annulus. Ascospores fusiform, hyaline, transversely septate.

##### Habitat and geographical distribution.

Saprobes on dead leaves of *Freycinetia* spp. (Pandanaceae) and decaying wood, known only in Australasia in New Caledonia and New Zealand (McKenzie 1991; this study).

##### Notes.

The genus *Chalarodes*, typified with *Cha. bisetis*, was erected for dematiaceous hyphomycetes observed on leaf litter of *Freycinetia* spp. in New Zealand and New Caledonia (McKenzie 1991). It is characterised by mononematous, simple, dark brown conidiophores with terminal monophialidic conidiogenous cells extending percurrently and hyaline, aseptate, cuneiform, obconical to obtriangular conidia with setulae, adhered in short basipetal chains. In the protologue (McKenzie 1991), the conidia were described only in the side view with two simple setulae at the apical end. Based on the examination of newly collected material, the conidia have angular outline when viewed from above; they have (3–)4 corners with a setula inserted in each corner. Additionally, we observed sterile setae growing among the conidiophores or on the ascomatal wall. They resemble capitate hyphae of *Catenularia*, but the mucilaginous sheath around the apex was lacking.

To date, two species, *Cha. bisetis* and *Cha. obconica*, have been placed in *Chalarodes* (McKenzie 1991). A new species, *Cha. obpyramidata*, inhabiting decaying wood and originating from New Zealand is introduced below. The teleomorph-anamorph connection of *Chalarodes* is described for the first time. Based on the results of the phylogenetic study, *Cha. obpyramidata* is closely related to *Catenularia*.

#### Chalarodes obpyramidata

Taxon classificationFungiChaetosphaerialesChaetosphaeriaceae

Réblová sp. nov.

CDAC22E7-0887-54D7-A00B-EB39E3214F1A

839467

Fig. 8

##### Etymology.

*Pyramidatus* (L), pyramidal, prefix *ob*- (L), meaning reversely, inversely, referring to the conidial shape.

##### Type.

New Zealand – West Coast region • Westland district, Ross, Totara forest, Totara River valley; on decaying wood of a branch; 7 Mar. 2003; M. Réblová MR 2734/NZ 236 (***holotype***PDD 119363).

##### Description.

Colonies on natural substrate effuse, hairy, dark brown to black, mycelium partly superficial, partly immersed, brown; colonies composed of conidiophores and sometimes ascomata. ***Anamorph*.** Setae present, mostly associated with ascomata (see below). Conidiophores 195–360 × 5–7.5 μm, 7–8.5 μm wide above the base, mononematous, macronematous, solitary, erect, straight or flexuous, unbranched, thick-walled, dark brown, paler towards the apex. Conidiogenous cells 20–54 × 5–6.5(–8) μm tapering to 3.5–4.5 μm below the collarette, integrated, terminal, monophialidic, extending percurrently, cylindrical to cylindrical-lageniform, brown, producing conidia successively; collarettes 6–7.5 μm wide, 2.5–3(–4) μm deep, funnel-shaped, pale brown. Conidia 10.5–12 μm long, 8.5–12 μm wide, 2.5–3.5 μm wide at the basal hilum (mean ± SD = 11.2 ± 0.5 × 10.3 ± 1.0 × 2.9 ± 0.3 μm), obpyramidal, in side view cuneiform to obtriangular, with four corners when viewed from above, truncate at the basal scar, with straight or curved setulae inserted at each corner 5–8 μm long, aseptate, hyaline, thin-walled, smooth; formed singly, adhered in basipetal chains. ***Teleomorph*.** Ascomata 120–140 μm diam, 130–160 μm high, subglobose, dark brown to black, superficial, solitary or aggregated, subglobose, papillate, setose. Setae 37–157 × 3.5–5.5 μm, simple, straight, cylindrical, brown, pale brown towards the apex, extending percurrently, apical cell sterile, 3.5–4 μm wide, broadly rounded, pale brown to subhyaline, similar setae arise around ascomata on the substrate. Ostiole periphysate. Ascomatal wall fragile, carbonaceous, 20–24 μm thick, two-layered. Outer layer consisting of brown, polyhedral cells with opaque walls. Inner layer consisting of several rows of thin-walled, hyaline cells. Paraphyses 4–5 μm wide, tapering to ca. 2 μm, septate, hyaline, longer than the asci. Asci 95–114 × (9–)10–12 μm (mean ± SD = 103.5 ± 6.5 × 10.9 ± 1.1 µm), cylindrical-clavate, short-stipitate, apically narrowly rounded, ascal apex with a non-amyloid apical annulus ca. 3 μm wide, 2 μm high. Ascospores 18–22(–23) × 4–5 μm (mean ± SD = 20.4 ± 1.3 × 4.4 ± 0.4 µm), fusiform, hyaline, 1–3-septate, smooth, 2-seriate in the ascus.

**Figure 8.**
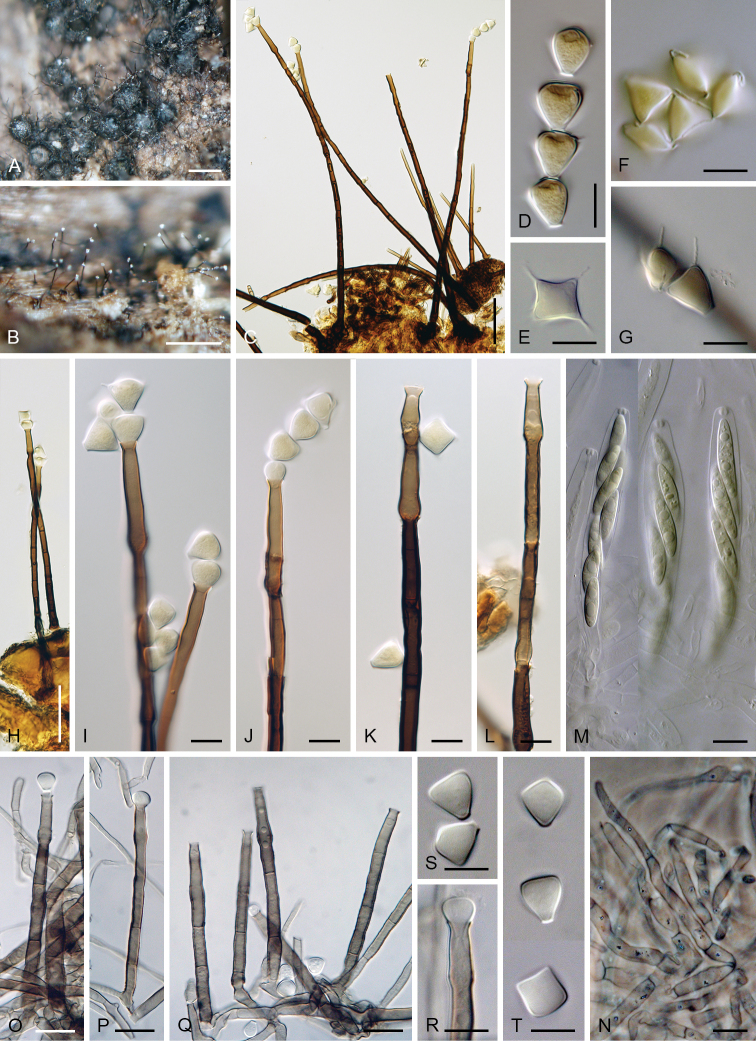
*Chalarodes obpyramidata***A** ascomata **B** colony composed of conidiophores **C, H, O–Q** conidiophores **D–G, S, T** conidia **I–L, R** upper parts of conidiophores with conidia **M** asci with ascospores **N** paraphyses. Images: PDD 119363 (**A–L**); PDD 119364 (**M–T**); on natural substrate (**A–N**); in PCA culture after 4 wk (**O–T**). Scale bars: 250 μm (**A, B**); 50 μm (**C, H**); 10 μm (**D–G, M, N, R–T**); 20 μm (**I–L, O–Q**).

##### Culture characteristics.

On PCA: colonies 7–10 mm diam in 14d, circular, raised, margin entire, velvety-lanose, brown to dark grey-brown with whitish-grey conidial masses, reverse black. Sporulation abundant at the centre of the colony.

Colonies on PCA effuse, mycelium subhyaline to pale brown, 2–3 μm wide. Setae absent. Conidiophores, conidiogenous cells and conidia similar to those from nature. Conidiophores 74–141 × (4.5–)5–6 μm, 5.5–6.5 μm wide above the base, paler brown and less septate than those from nature, erect, straight. Conidiogenous cells 18–40 × 4.5–5.5 μm tapering to 3.5–4 μm below the collarette, cylindrical, pale brown; collarettes 5–6 μm wide, 3.5–4 μm deep, pale brown. Conidia 8–10(–11) μm long, 8–9(–10) μm wide, 2–2.5 μm wide at the hyaline basal hilum (mean ± SD = 9.7 ± 0.9 × 8.4 ± 0.6 × 2.0 ± 0.1 μm), cuneiform to obpyramidal, truncate at the basal scar, setulae not observed, aseptate, hyaline, thin-walled, smooth, formed basipetally in chains.

##### Other specimen examined.

New Zealand – West Coast region • Buller district, Victoria Forest Park, Reefton, Big River Inanganua track; on decaying wood of *Nothofagus* sp. (associated with *C. novae-zelandiae*PDD 119362 and *Zanclospora falcata*PDD 119365); 6 Mar. 2003, M. Réblová MR 2724/ NZ 225 (PDD 119364).

##### Habitat and geographical distribution.

Saprobe on decaying wood, known only in New Zealand.

##### Notes.

In the size of conidia, our species appears intermediate between *Cha. bisetis* and *Cha. obconica* (McKenzie 1991). *Chalarodes bisetis* differs from *Cha. obpyramidata* in conidia longer and narrower at the apical end, (9.5–)12–14(–15) × 4.5–6(–9) μm, while *Ch. obconica* possesses conidia slightly shorter (8–)9–10.5(–11) μm and narrower at the basal hilum 1.75–2 μm.

#### Fuscocatenula

Taxon classificationFungiChaetosphaerialesChaetosphaeriaceae

Réblová & A.N. Mill. gen. nov.

1E102A38-67F9-5A33-8F5D-5470F29B2518

839468

##### Etymology.

*Fuscus* (L) dark, brown, dusky, *catenula* (L), a little chain, referring to pigmented conidia in chains.

##### Type species.

*Fuscocatenula submersa* (Z.L. Luo, K.D. Hyde & H.Y. Su) Réblová & A.N. Mill.

##### Description.

Colonies effuse, hairy, brown, mycelium partly immersed, partly superficial. ***Anamorph*.** Conidiophores macronematous, mononematous, solitary, erect, unbranched, brown to dark brown, thick-walled, paler and thinner-walled towards the apex. Conidiogenous cells integrated, terminal, monophialidic, extending percurrently, cylindrical to lageniform, brown; collarettes funnel-shaped, brown. Conidia cuneiform to obovoid, broadly rounded apically, truncate at the base, aseptate, hyaline when young, pale brown at maturity, with protracted maturation, smooth, formed in a basipetal chain. ***Teleomorph*.** Unknown. (Description partly adapted from Li et al. 2017; Luo et al. 2019).

##### Habitat and geographical distribution.

Members of the genus are saprobes on decaying plant matter in terrestrial and freshwater environments, known only in Asia in China.

##### Notes.

*Fuscocatenula* is proposed as a segregate genus for fungi distantly related from *Catenularia* (Fig. 2), although morphologically similar. Conidia of *Fuscocatenula* are obovoid with a truncate base, lack an angular outline and small, circular, thin-walled pale areas in corners that are present in *Catenularia*. Conidia have a protracted maturation; at first they are hyaline and only later become pale brown, while still attached in a chain. Sometimes the chain consists of hyaline conidia with only one or a few mature pigmented conidia (Li et al. 2017: fig. 1; Luo et al. 2019: fig. 52). In *Catenularia*, conidia are also hyaline when young but mature soon and when released from the conidiogenous locus they are usually pigmented. Since *Catenularia* also includes species lacking capitate hyphae, this character alone is not reliable in the distinction of *Fuscocatenula* from *Catenularia*.

Two species are accepted in the genus. Li et al. (2017) introduced *Catenularia variegata* for a foliicolous species from China and Luo et al. (2019) described *Chaetosphaeria submersa* for a dematiaceous hyphomycete from submerged wood in Thailand. Both species are similar and reminiscent of *Catenularia*. In the phylogenetic analysis based on ITS-28S sequences, relationship of *Ch. submersa* and *Catenularia* was not supported. Molecular data of *C. variegata* are not available. Based on a detailed comparison of original descriptions and illustrations of both species we conclude that *C. variegata* is congeneric with *Ch. submersa*. Therefore, *C. variegata* is excluded from *Catenularia* and both species are transferred to the new genus *Fuscocatenula*.

#### Fuscocatenula submersa

Taxon classificationFungiChaetosphaerialesChaetosphaeriaceae

(Z.L. Luo, K.D. Hyde & H.Y. Su) Réblová & A.N. Mill. comb. nov.

02B6B392-5CCD-5B4C-989F-F492AE1DA004

839469

Basionym. Chaetosphaeria submersa Z.L. Luo, K.D. Hyde & H.Y. Su, Fungal Divers. 99: 585. 2019.

##### Habitat and geographical distribution.

Saprobe on submerged decaying wood in stream, known only in China (Luo et al. 2019).

##### Notes.

The species is characterised by conidiophores 380–596(–691) μm × 15–21 μm and cuneiform, pale brown conidia 21–27 × 12–14 μm. The size of these structures clearly distinguishes *F. submersa* from the small-spored *F. variegata* with shorter conidiophores (Luo et al. 2019).

#### Fuscocatenula variegata

Taxon classificationFungiChaetosphaerialesChaetosphaeriaceae

(H.H. Li & X.G. Zhang) Réblová & A.N. Mill. comb. nov.

1FCC2EAE-3372-5D96-93A8-FA44DD7FEA29

839470

Basionym. Catenularia variegata H.H. Li & X.G. Zhang, Mycotaxon 132: 621. 2017.

##### Habitat and geographical distribution.

Saprobe on dead stems of an unidentified broadleaf tree, known only in China (Li et al. 2017).

##### Notes.

*Fuscocatenula variegata* resembles *F. submersa* but differs in shorter conidia 8.5–11 × 5.5–7.5 μm and shorter conidiophores 150–270 × 4.5–8 μm (Li et al. 2017).

#### Nawawia antennata

Taxon classificationFungiChaetosphaerialesChaetosphaeriaceae

Réblová sp. nov.

604D1BAD-5152-59C1-8374-B8F5133AD7C4

839471

Fig. 9

##### Etymology.

*Antennatus* (L) meaning ‘having antenna(s)’, referring to the presence of conidial appendages resembling insect antennas.

##### Type.

Thailand – Nakhon Nayok Province • Khao Yai National park, Phakrajai trail, on decaying wood and bark of a twig; 17 Aug. 2001; M. Réblová & N. Hywel-Jones M.R. 2056/TH 219 (PRA-20374).

##### Description.

Colonies on natural substrate effuse, hairy, dark brown, mycelium partly superficial, partly immersed, brown. ***Anamorph*.** Conidiophores forming two distinct layers; conidiophores of the upper layer 142–282 μm long, conidiophores of the lower layer 44–90 μm long, 5–6 μm wide, 6–8.5 wide above the base, basal cell bulbose with dark brown, thick-walled stromatic cells around the base, mononematous, macronematous, solitary or fasciculate in a group of 2–6, erect, straight or flexuous, unbranched, thick-walled, dark brown, paler towards the apex. Conidiogenous cells 19.5–29 × 5.5–7.5(–8) μm tapering to 3–5 μm below the collarette, integrated, terminal, monophialidic, extending percurrently, subcylindrical to lageniform, pale brown; collarettes 5.5–6.5 μm wide, 1.5–2.5 μm deep, funnel-shaped, pale brown. Conidia 14–17(–18) μm long, 11–14.5(–15.5) μm wide, 2.5–3.5 μm wide at the basal hilum (mean ± SD = 15.5 ± 1.2 × 12.9 ± 1.7 × 2.9 ± 0.3 μm), turbinate to obpyramidal, in side view cuneiform to obtriangular, truncate at the basal scar, flattened to slightly concave at the apical end, with (3–)4 corners when viewed from above, aseptate, hyaline, thin-walled, smooth, with simple setulae inserted at each corner, 17–43 μm long, 7.5–20 μm long when the ends are coiled, conidia accumulate in slimy droplets. ***Teleomorph*.** Not observed.

**Figure 9.**
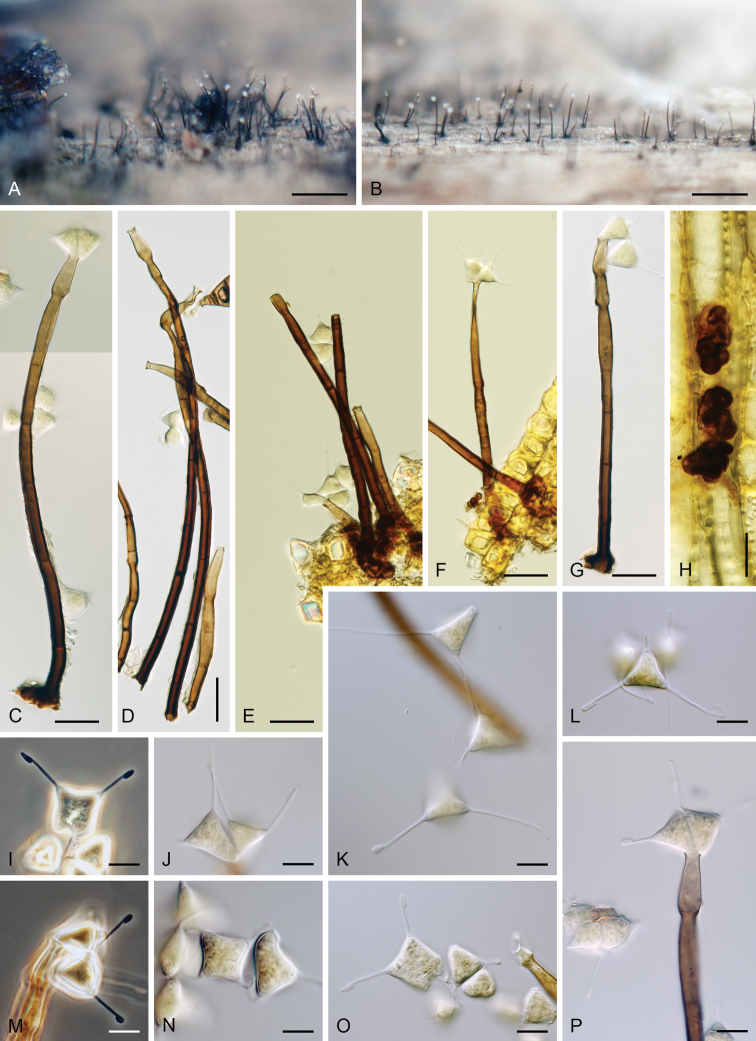
*Nawawia antennata* (PRA-20374 holotype) **A, B** colony **C–G** conidiophores **H** stromatic cells **I–P** conidia. Images: on natural substrate (**A–P**). Scale bars: 250 μm (**A, B**); 20 μm (**C, F, G**); 25 μm (**D, E**); 10 μm (**H–N**).

##### Habitat and geographical distribution.

Saprobe on decaying wood, known only in Thailand.

##### Notes.

We were unsuccessful in obtaining *N. antennata* in axenic culture. The species exhibits diagnostic characteristics of *Nawawia* such as pigmented, mononematous conidiophores with stromatic cells around the base, terminal monophialides extending percurrently and hyaline, aseptate, obtriangular conidia with an angular outline and several simple setulae at the apex. Conidia accumulate in a slimy head. Conidiophores forming two distinct layers were also documented in *N. quadrisetulata* (Goh et al. 2014: figs 2, 3).

Among *Nawawia* species, *N. antennata* is well distinguished by coiled appendages and the size of conidia. *Nawawia quadrisetulata* is similar to the new species in conidia with mostly four angles at the apex but differs in larger conidia (30–37.5 × 22.5–32.5 μm) with longer setulae (30–57.5 μm). *Nawawia antennata* resembles *N. filiformis* (Marvanová 1980) but the latter species has conidia wider at the apex (14–18 μm) and straight appendages.

## Discussion

In this study, we have reviewed the generic concept of *Catenularia* and its relationships with morphologically similar genera with catenate conidia using molecular and phenotypic data. The conidial characteristics, such as the colour at maturity, the outline in transverse section and presence or absence of the setulae are the main taxonomic criteria at the generic rank for distinguishing between *Catenularia*, *Chalarodes* and *Fuscocatenula*. Their conidia are formed successively; they are solitary and adhere in basipetal chains. These genera are compared with *Nawawia*, *Obeliospora* and *Phialosporostilbe*, which have similar conidia in slimy heads.

Although molecular DNA data of *C. cupulifera* are not available, four other morphologically similar species accepted in *Catenularia* were included in the analysis of ITS and 28S sequence data. *Catenularia* was resolved as a monophyletic strongly supported clade. Phylogenetic analysis indicates that *Chaetosphaeria* (Tulasne and Tulasne 1863), based on *Ch. innumera* with the *Chloridium botryoideum* anamorph (Gams and Holubová-Jechová 1976), is a phylogenetically distinct genus (Fig. 2). Therefore, *Catenularia* is proposed as the generic name for a morphologically well-delimited group of species whose teleomorphs were previously attributed to *Chaetosphaeria*. The correct epithet of the type species of *Catenularia* is ‘*cupulifera*’ based on *Sphaeria cupulifera* 1871, the earliest available epithet at the species rank; *C. cuneiformis* 1877 and *C. simplex* 1886 are reduced to synonymy. *Catenularia* is delimited to fungi with pigmented conidiophores arising singly or in tufts, usually accompanied by capitate hyphae, terminal monophialidic conidiogenous cells extending percurrently and flared collarettes. Conidia are pigmented, aseptate, thick-walled, formed successively from the conidiogenous locus and usually adhere in chains. They are cuneiform to rounded-obconic in side view with several blunt corners when viewed from above, each with a small, thin-walled, pore-like area. The associated teleomorphs have perithecial ascomata, unitunicate 8-spored asci, persistent paraphyses and hyaline, fusiform, transversely septate ascospores. *Catenularia* grows on decaying bamboo culms and bark and wood of various hosts in terrestrial or freshwater habitats worldwide.

Eleven species are accepted in *Catenularia*, four of which have been verified with molecular DNA data. One of the accepted species, *C. elsikii*, is a fossil fungus. The conidia were preserved in a sample of fossil wood, dated to the Miocene, found in the United Kingdom (Pound et al. 2019). The substrate indicates a similar habitat as in the current species. Microscopic fossil fungi are difficult to identify, especially when only spores or fragments of reproductive structures are preserved (Taylor et al. 2015). Fortunately, *Catenularia* conidia represent a distinctive morphotype, which allows reliable identification. The majority of species of the Chaetosphaeriaceae have hyaline, thin-walled conidia and ascospores, which will likely disintegrate in the fossilized samples. On the other hand, thick-walled and heavily pigmented fungal reproductive structures are randomly present in fossil material (Pound et al. 2019). Apart from *Catenularia*, *Adautomilanezia* (Crous et al. 2016), *Ellisembia*, *Stanjehughesia* (Subramanian 1992), and *Sporoschisma* (Berkeley and Broome 1871; Hughes 1966) of the Chaetosphaeriaceae also have thick-walled and melanised conidia that may occur in fossil material or palynological preparations.

Hughes (1965) suggested that conidia of *Catenularia* may germinate through the inconspicuous, thin-walled areas in the cell wall in corners. In the newly recorded specimens of *C. novae-zelandiae*, we observed several conidia with rudimentary hyaline appendages growing from these pore-like areas (Fig. 7K). This feature has not been recorded in any other *Catenularia* species. However, we rule out the possibility that these appendages are germinating tubes after comparing the figure in Luo et al. (2019: figure 47l) depicting germinating conidium. The presence of rudimentary conidial appendages in *Catenularia* may reflect its newly revealed phylogenetic relationship.

In the ITS-28S phylogeny, *Chalarodes* was shown as a sister to *Catenularia* with high statistical support. Their close relationship is also supported by similar morphologies. *Chalarodes* differs from *Catenularia* in conidia that are hyaline at maturity and have simple setulae at the apical end. Although McKenzie (1991) described conidia of two *Chalarodes* species from the side view only, examination of our material revealed that the conidia are turbinate to obpyramidal with an angular outline. The discovery of rudimentary setulae in *C. novae-zelandiae* provides a new perspective on this characteristic. Although setulae persist in *Chalarodes*, the appendages in *Catenularia* were lost during evolution or never evolved, except in the discovered case. However, the systematic placement of *C. novae-zelandiae* has yet to be confirmed with DNA sequence data. Our observations of *Cha. obpyramidata* in culture (Fig. 8O–T) correspond to those of Marvanová (1980) on *Nawawia filiformis*. In both species, conidia that formed in culture lack setulae.

*Fuscocatenula* is proposed for fungi similar to *Catenularia* and readily distinguished by pigmented conidia with protracted maturation, round in transverse section, lacking minute pore-like areas at the apical end, and the absence of capitate hyphae. In the phylogenetic analysis, *Fuscocatenula* was shown as a separate lineage, related to several *Chaetosphaeria* with hyaline or slightly pigmented conidia formed singly or in chains (Gams and Holubová-Jechová 1976). Its closest relatives are *Ch. mangrovei* with an unknown conidial state, and *Ch. innumera*. *Chloridium botryoideum*, the anamorph of *Ch. innumera*, forms hyaline ellipsoidal conidia arranged in imbricate chains or large heads on sympodially elongating conidiogenous cells. *Phaeostalagmus cyclosporus* and two *Chaetosphaeria* species with *Chloridium* anamorphs are shown as a sister subclade to *Fuscocatenula*. *Chloridium clavaeforme* and *Chl. phaeophorum* belong to the section Gongromeriza and resemble *Fuscocatenula* in slightly pigmented, short-cuneiform or dacryoid conidia forming chains or slimy droplets. *Phaeostalagmus*, on the other hand, represents a different phenotype. Its conidiophores are branched with lateral or terminal monophialides producing hyaline, ellipsoidal conidia in slimy heads.

Capitate hyphae (Hughes 1949) are a prominent characteristic that occurs in several members of the Chaetosphaeriaceae. They accompany conidiophores of *Catenularia* and *Sporoschisma*; they are scattered on the substrate or more frequently grow in tufts among the conidiophores or on ascomata of their teleomorphs. Capitate hyphae also occur on ascomata of *Ch. capitata*, the teleomorph of *Exserticlava vasiformis*, and *Ch. conirostris* (Sivanesan and Chang 1995; Fernández and Huhndorf 2005). Similar setae with a swollen apical cell but without the mucilaginous cap were observed on and around ascomata of the teleomorph of *Cha. obpyramidata* (this study). The presence of analogous structures have been described in the teleomorph of *Stanjehughesia* (Réblová 1999); they cover ascomata and their apical part, separated by a septum, is formed by an amorphous, subhyaline, clavate to almost triangular globule. All these genera, except for *Sporoschisma*, clustered as members of a robust clade at the base of the family tree.

Because of its mononematous conidiophores and hyaline, tetrahedral conidia with setulae arranged in corners at the apical end, *Chalarodes* appears similar to *Nawawia* (Marvanová 1980). *Nawawia* encompasses aero-aquatic fungi that form effuse, hairy colonies on decaying wood, bamboo culms and petioles. It is distinguished from *Chalarodes* by conidia that do not adhere in chains; instead they are single or accumulate in heads at the tip of the conidiogenous cells. Conidiophores often have small stromatic hyphal cells around the base. *Nawawia* accommodates five species of which only four, namely *N. antennata*, *N. filiformis*, *N. quadrisetula*, *N. sasae-kurilensis*, correspond to the generic concept based on *N. filiformis* (Marvanová 1980; Mel’nik and Hyde 2006; Goh et al. 2014; this study). The new species *N. antennata* resembles *N. quadrisetula* (Goh et al. 2014) in characters of conidiophores and conidia but differs in that the conidia are smaller and the setulae are coiled. Unfortunately, living culture or molecular data are not available to confirm its relationships. *Nawawia oviformis* (Peng et al. 2016) does not fit the circumscription of the genus; it has conidia with a round outline in transverse section with setulae arranged irregularly over the whole surface. These characteristics are typical of *Bahusutrabeeja* (Subramanian and Bhat 1977) and *N. oviformis* would be better placed in this genus. In the ITS-28S phylogenetic tree (Fig. 2), *Nawawia* and *Bahusutrabeeja* form separate lineages. Three species originally attributed to *Nawawia* have been reclassified and placed in other genera as *Neonawawia malaysiana* (Yang et al. 2018), *Obeliospora nitida* (Cantillo-Pérez et al. 2018) and *Phialosporostilbe dendroidea* (Yang et al. 2018). *Neonawawia* is particularly interesting by its formation of sporodochial conidiomata and hyaline to light brown conidiophores; it resembles *Nawawia* only in the characteristics of conidia. Based on phylogenetic evidence, its placement has been confirmed outside the Chaetosphaeriaceae (Yang et al. 2018).

Hyaline, turbinate conidia with an angular outline and apical setulae represent an uncommon morphotype in the Chaetosphaeriaceae. Apart from *Chalarodes* and *Nawawia*, similar conidia borne on monophialides occur only in species of *Phialosporostilbe*. The latter genus is distantly related to both genera and is distinguished by synnematous conidiophores associated with setae, conidial setulae occasionally formed at the base and a chloridium-like synanamorph (Mercado Sierra and Mena Portales 1985; Bhat and Kendrick 1993). The synnemata are indeterminate and although in most species the stalk is formed by compact conidiophores that climb upwards along the seta and diverge at their fertile apices, the arrangement of conidiophores of *P. gregariclavata* (Shirouzu and Harada 2004) is unusual within the genus. The central setiform conidiophore is accompanied by a group of shorter, parallel conidiophores that are solitary or tightly adhering to each other and may fuse. Therefore, the conidiophores of *P. gragariclava* may be interpreted as a poorly developed synnemata (Shirouzu and Harada 2004: fig. 10). In the characters of conidiophores, *P. gregariclavata* resembles members of *Nawawia*.

In characteristics of conidia, *Chalarodes*, *Nawawia* and *Phialosporostilbe* are comparable with *Obeliospora*, whose systematic placement remains unknown. The genus was emended by Cantillo-Pérez et al. (2018) and is readily distinguished by the absence of stromatic hyphal cells, and the presence of dark acute setae accompanied by monilioid conidiophores with terminal doliiform conidiogenous cells and flared, cup- or funnel-shaped collarettes. The conidia vary in shape ranging from round-tetrahedral, conical, pyramidal to subglobose and are hyaline, although in some species older conidia become light brown.

Although we emphasised characteristics of conidia in chains or heads to support delimitation of *Catenularia*, *Chalarodes* and *Nawawia*, we should look at this diagnostic trait with caution. For example, in *C. minor* conidia adhere in chains but in older parts of the colony conidia may form clusters. The chains break into smaller fragments, which appear as a cluster at the tip of the conidiogenous cell. In microscopic preparation, the chains readily break up into solitary conidia (Fig. 6E, L). A similar variability occurs in *Phialosporostilbe*. Although the majority of species have conidia arranged in slimy heads, the conidia of *P. catenata* form chains (Sureshkumar et al. 2005). Réblová et al. (2011) discussed this phenomenon using the example of *Monilochaetes camelliae* observed with an ESEM (Environmental Scanning Electron Microscope). The authors showed that there is a continuum from conidial chains to slimy heads on the phialides in culture. *Chloridium* is another example, e.g. *Chl. clavaeforme* and *Chl. virescens*, in which chains, cirrhi, and slimy heads can all be observed in one species in culture (Gams and Holubová-Jechová 1976; pers. obs.). It is apparently caused by the osmolarity of the medium that may affect the proportion between chains and heads.

The present investigation contributes to the knowledge of *Catenularia* and similar fungi with catenate conidia placed in the Chaetosphaeriaceae. Sampling of other species in the genera *Catenularia*, *Chalarodes*, *Nawawia* and *Phialosporostilbe*, which have not yet been verified by molecular data, are needed to address their systematic placement.

## Supplementary Material

XML Treatment for Catenularia

XML Treatment for Catenularia angulospora

XML Treatment for Catenularia catenulata

XML Treatment for Catenularia cubensis

XML Treatment for Catenularia cupulifera

XML Treatment for Catenularia elsikii

XML Treatment for Catenularia kalakadensis

XML Treatment for Catenularia longispora

XML Treatment for Catenularia macrospora

XML Treatment for Catenularia malabarica

XML Treatment for Catenularia minor

XML Treatment for Catenularia novae-zelandiae

XML Treatment for Chalarodes

XML Treatment for Chalarodes obpyramidata

XML Treatment for Fuscocatenula

XML Treatment for Fuscocatenula submersa

XML Treatment for Fuscocatenula variegata

XML Treatment for Nawawia antennata

## Supplementary materials

Supplementary material 1 Table S1. Taxa, isolate information and accession numbers for sequences retrieved from GenBank Data type molecular data Explanation note New sequences determined for this study and taxonomic novelties are given bold https://binary.pensoft.net/file/554160This dataset is made available under the Open Database License (http://opendatacommons.org/licenses/odbl/1.0/). The Open Database License (ODbL) is a license agreement intended to allow users to freely share, modify, and use this Dataset while maintaining this same freedom for others, provided that the original source and author(s) are credited.Martina Réblová1, Jana Nekvindová2, Andrew N. Miller
