# Supplementary material for: Phylogeny and taxonomy of Catenularia and similar fungi with catenate conidia
Source: MycoKeys. 2021 Jun 11;81:1–44. doi: 10.3897/mycokeys.81.67785 (PMC8213683; doi:10.3897/mycokeys.81.67785)
Supplement: Supplementary material 1 — Table S1. Taxa, isolate information and accession numbers for sequences retrieved from GenBank [file mycokeys-81-001-s001.pdf]

Table S1. Taxa, isolate information and accession numbers for sequences retrieved from GenBank. New sequences determined for this study and taxonomic novelties are given bold.

Taxon	Strain	Status	Country	Host	Substrate	GenBank accessions	
						ITS	28S
Adautomilanezia caesalpiniae	CC-LAMIC 102/12	T	Brazil	*Caesalpina echinata*	wood	KX821777	KU170671
Achrochaeta talbotii	ICMP 15161		New Zealand	unidentified	decaying wood	MT454480	MT454495
Anacacumisporium appendiculatum	HMAS 245593	T	China	broad-leaved tree	dead stems	KP347129	KT001553
Bahusutrabeeja dwaya	CBS 261.77	T	India	*Coffea arabica*	dead twig	MH861059	MH872829
Brunneodinemasporium brasiliense	CBS 112007	T	Brazil	unidentified	decaying leaf	JQ889272	JQ889288
Brunneodinemasporium jonesii	GZCC 16-0050	T	China	unidentified	decaying wood	KY026058	KY026055
Cacumisporium capitulatum	FMR 11339		Spain	unidentified	decaying wood	HF677176	HF677190
Calvolachnella guaviyunis	CBS 134695	T	Uruguay	*Myrcianthes pungens*	bark	KJ834524	KJ834525
Catenularia cubensis	S.M.H. 3258		Costa Rica	unidentified	decaying wood	**MW987826**	AF466067
Catenularia angulospora	MFLUCC 18-1331[#]		China	unidentified	submerged wood	MK828638	MK835840
Catenularia catenulata	S-298		China	unidentified	submerged wood	—	MK835839
Catenularia catenulata	DLUCC 0891	T	China	unidentified	submerged wood	MK828637	MK835838
Catenularia minor	PRM 900544*	T	Thailand	unidentified	bamboo culm	**MW987827**	**MW987822**
Chaetosphaeria conirostris	S.M.H. 2183	T	Costa Rica	unidentified	decaying wood	—	AF466066
Chaetosphaeria curvispora	ICMP 18255		New Zealand	unidentified	decaying wood	—	GU180636
Chaetosphaeria fusiformis	CBS 101429		Czech Republic	*Abies alba*	decaying bark	AF178554	AF178554
Chaetosphaeria innumera	M.R. 1175		Czech Republic	*Fagus sylvatica*	decaying wood	AF178551	AF178551
Chaetosphaeria lignomollis	S.M.H. 3015	T	Puerto Rico	unidentified	decaying wood	EU037896	AF466073
Chaetosphaeria mangrovei	MCD 069	T	Thailand	mangrove	decaying wood	MG813821	MG813820
Chaetosphaeria metallicans	PDD 92539	T	New Zealand	*Nothofagus* sp.	decaying wood	EU037893	EU037899
Chaetosphaeria myriocarpa	CBS 264.76	N	The Netherlands	unidentified	decaying wood	AF178552	AF178552
Chaetosphaeria pygmaea	M.R. 1365		Czech Republic	*Fagus sylvatica*	decaying wood	AF178545	AF178545
Chalarodes obpyramidata	PDD 119364	T	New Zealand	*Nothofagus* sp.	wood	**MW987828**	**MW987823**
Chloridium caesium	CBS 102339		Austria	*Salix cinerea*	decaying wood	AF178564	AF178564
Chloridium gonytrichii	CBS 195.60		South Africa	unidentified	unknown	MH857954	MH869503
Chloridium virescens	CBS 152.53		France	*Acer* sp.	unknown	MH857142	MH868678
Codinaea paniculata	CBS 145098	T	France	unidentified	submerged wood	MT118230	MT118201
Codinaeopsis gonytrichodes	CBS 593.93		Japan	unidentified	decaying plant material	AF178556	AF178556
Conicomyces pseudotransvaalensis	HHUF 29956	T	Japan	*Machilus japonica*	dead twig	LC001710	LC001708
Cryptophiale hamulata	MFLUCC 18-0098	E	Thailand	unidentified	decaying leaf	—	MG386756
Cryptophiale udagawae	GZCC 18-0047		China	unidentified	decaying wood	MN104608	MN104619
Cryptophialoidea fasciculata	MFLU 18-1499		Thailand	unidentified	submerged wood	MH758195	MH758208
Dendrophoma cytisporoides	CBS 144107		Germany	*Buxus sempervivens*	decaying bark	MT118234	MT118205
Dictyochaeta callimorpha	ICMP 15155		New Zealand	unidentified	decaying wood	MT454484	MT454499
Dictyochaeta fuegiana	ICMP 15153	T	New Zealand	unidentified	decaying wood	MT454487	EF063574
Dictyochaeta querna	CBS 145503		Czech Republic	*Quercus cerris*	acorn	MT454489	MT454503
Dictyochaeta terminalis	GZCC 18-0085	T	China	unidentified	decaying leaves	MN104613	MN104624

Dinemasporium cruciferum	HHUF 30001		Japan	*Arundo donax*	unknown	AB900895	AB934039
Dinemasporium decipiens	CBS 592.73		Suriname	n/a	soil under *Elaeis guineensis*	JQ889275	JQ889291
Dinemasporium pseudoindicum	CBS 127402	T	USA	n/a	soil of tallgrass prairie	JQ889277	JQ889293
Ellisembia aurea	CBS 144403	T	France	*Sambucus nigra*	decaying wood	MH836375	MH836376
Ellisembia folliculata	CBS 101317		France	*Salix* sp.	decaying wood	—	AF261071
Eucalyptostroma eucalypti	CBS 142074	T	Malaysia	*Eucalyptus pellita*	leaf spots	KY173408	KY173500
Exserticlava vasiformis	TAMA 450		Japan	unidentified	plant debris	—	AB753846
Fuscocatenula submersa	MFLUCC 18-1342	T	China	unidentified	submerged wood	MK828634	MK835835
Fuscocatenula submersa	S-844		China	unidentified	submerged wood	—	MK835834
Infundibulomyces cupulatus	BCC 11929	T	Thailand	*Lagerstroemia* sp.	dead leaf	EF113976	EF113979
Infundibulomyces oblongisporus	BCC 13400	T	Thailand	unidentified, angiosperm	leaf litter	EF113977	EF113980
Kionochaeta castaneae	GZCC 18-0025	T	China	*Castanea mollissima*	decaying seed shell	MN104610	MN104621
Kionochaeta microspora	GZCC 18-0036	T	China	unidentified	decaying wood	MN104607	MN104618
Kionochaeta ramifera	MUCL 39164		Cuba	unidentified	leaf	MW144421	MW144404
Menispora caesia	CBS 144659		Czech Republic	unidentified	decaying wood	MW984578	MW984560
Menispora ciliata	CBS 122131	T	Czech Republic	*Acer campestre*	decaying wood	EU488736	MH874726
Menispora tortuosa	DAOM 231154		n/a	unidentified	n/a	KT225527	AY544682
Menisporopsis breviseta	GZCC 18-0071	T	China	unidentified	decaying leaves	MN104612	MN104623
Menisporopsis dushanensis	GZCC 18-0084	T	China	unidentified	decaying leaves	MN104615	MN104626
Menisporopsis theobromae	MFLUCC 15-0055		Thailand	unidentified	submerged decaying wood	KX609957	KX609954
Nawawia filiformis	MFLUCC 17-2394		Thailand	unidentified	decaying wood	MH758196	MH758209
Neopseudolachnella acutispora	MAFF 244358	T	Japan	*Pleioblastus chino*	dead twigs	AB934065	AB934041
Neopseudolachnella magnispora	MAFF 244359	T	Japan	*Sasa kurilensis*	dead twigs	AB934066	AB934042
Paliphora intermedia	CBS 896.97	I	Australia	unidentified	leaf litter	MH862682	EF204501
Paragaeumannomyces garethjonesii	MFLUCC 15-1012	T	Thailand	Fabaceae	seed pod	KY212751	KY212759
Paragaeumannomyces panamensis	S.M.H. 3596	T	Panama	unidentified	decaying wood	AY906948	MT118218
Paragaeumannomyces rubicundus	S.M.H. 3221	T	Costa Rica	unidentified	decaying wood	MT118242	MT118224
Paragaeumannomyces sabinianus	ILLS121384	T	USA	unidentified	decaying wood	MT118243	MT118225
Phaeostalagmus cyclosporus	CBS 663.70		The Netherlands	*Quercus* sp.	decaying bark	MH859892	MH871680
Phialosporostilbe scutiformis	MFLUCC 17-0227	T	China	unidentified	submerged decaying wood	MH758194	MH758207
Polynema podocarpi	CBS 144415	T	New Zealand	*Podocarpus totara*	unknown	MH327797	MH327833
Pseudodinemasporium fabiforme	CBS 140010		Malaysia	*Acacia mangium*	leaf spots	KR611889	KR611906
Pseudolachnea fraxini	CBS 113701	T	Sweden	*Fraxinus excelsior*	unknown	JQ889287	JQ889301
Pseudolachnea hispidula	MAFF 244365		Japan	*Morus bombycis*	dead twig	AB934072	AB934048
Pseudolachnella asymmetrica	MAFF 244366	T	Japan	*Phyllostachys nigra* var. *henonis*	dead twig	AB934073	AB934049
Pseudolachnella scolecospora	MAFF 244379		Japan	*Sasa* sp.	dead twigs	AB934086	AB934062
Pyrigemmula aurantiaca	CBS 126743	T	Hungary	*Vitis vinifera*	bark	HM241692	HM241692
Rattania setulifera	GUFCC 15501	T	India	*Calamus thwaitesii*	leaves	GU191794	HM171322
Sporoschisma hemipsilum	MUCL 56487		Martinique	unidentified	wood	**MW987829**	**MW987824**
Sporoschisma longicatenatum	MFLUCC 16-0180	T	Thailand	unidentified	submerged decaying wood	KX505871	KX358077
Sporoschisma mirabile	CBS 144794		France	*Alnus glutinosa*	submerged wood	**MW987830**	**MW987825**

Sporoschisma taitense	KUMCC 15-0241		China	unidentified	submerged wood	KX455865	KX455858
Stanjehughesia hormiscioides	CBS 102664		Ukraine	*Fagus sylvatica*	decaying wood	—	AF261069
Striatosphaeria castanea	CBS 145352	T	French Guinea	woody liana	decaying periderm	MT118244	MT118229
Striatosphaeria codinaeophora	M.R. 1230		Puerto Rico	*Dacryodes excelsa*	decaying wood	AF178546	AF178546
Tainosphaeria crassiparies	S.M.H. 1934	T	Puerto Rico	*Hymenaea* sp.	seed pod	—	AF466089
Tainosphaeria jonesii	GZCC 16-0065	P	China	unidentified	submerged decaying wood	KY026060	KY026057
Tainosphaeria siamensis	MFLUCC 15-0607	T	Thailand	unidentified	submerged decaying wood	KX609956	KX609953
Thozetella fabacearum	MFLUCC 15-1020	T	Thailand	Fabaceae	seed pod	KY212754	KY212762
Thozetella nivea	n/a		unknown	unidentified	unknown	EU825201	EU825200
Thozetella tocklaiensis	CBS 378.58	T	India	*Camellia sinensis*	decaying flower	MH857817	MH869349
Tracylla aristata	CBS 141404	E	Australia	*Eucalyptus regnans*	leaf	KX306770	KX306795
Tracylla eucalypti	CBS 144429	T	Colombia	*Eucalyptus urophylla*	spots on living leaves	MH327810	MH327846
Zanclospora iberica	CBS 130426	T	Spain	unidentified	decaying wood	KY853480	KY853544
Zanclospora novae-zelandiae	ICMP 15781	E	New Zealand	*Fuscospora cliffortioides*	decaying wood	MW144429	MW144411
Zanclospora xylophila	ICMP 22737	T	New Zealand	unidentified	decaying wood	MW144437	MW144417

Notes:
T, E, I, N and P denote ex-type, ex-epitype, ex-isotype, ex-neotype and ex-paratype strains.
Catenularia cubensis fide Luo et al. (2019)
* holotype of *Chaetosphaeria trianguloconidia*
